# Cell type-specific mechanisms of information transfer in data-driven biophysical models of hippocampal CA3 principal neurons

**DOI:** 10.1371/journal.pcbi.1010071

**Published:** 2022-04-22

**Authors:** Daniele Linaro, Matthew J. Levy, David L. Hunt

**Affiliations:** 1 Dipartimento di Elettronica, Informazione e Bioingegneria (DEIB), Politecnico di Milano, Milan, Italy; 2 Center for Neural Science and Medicine, Cedars-Sinai Medical Center, Los Angeles, California, United State of America; 3 Department of Neurosurgery, Cedars-Sinai Medical Center, Los Angeles, California, United State of America; 4 Department of Neurology, Cedars-Sinai Medical Center, Los Angeles, California, United State of America; 5 Department of Biomedical Sciences, Cedars-Sinai Medical Center, Los Angeles, California, United State of America; National Research Council, ITALY

## Abstract

The transformation of synaptic input into action potential output is a fundamental single-cell computation resulting from the complex interaction of distinct cellular morphology and the unique expression profile of ion channels that define the cellular phenotype. Experimental studies aimed at uncovering the mechanisms of the transfer function have led to important insights, yet are limited in scope by technical feasibility, making biophysical simulations an attractive complementary approach to push the boundaries in our understanding of cellular computation. Here we take a data-driven approach by utilizing high-resolution morphological reconstructions and patch-clamp electrophysiology data together with a multi-objective optimization algorithm to build two populations of biophysically detailed models of murine hippocampal CA3 pyramidal neurons based on the two principal cell types that comprise this region. We evaluated the performance of these models and find that our approach quantitatively matches the cell type-specific firing phenotypes and recapitulate the intrinsic population-level variability in the data. Moreover, we confirm that the conductance values found by the optimization algorithm are consistent with differentially expressed ion channel genes in single-cell transcriptomic data for the two cell types. We then use these models to investigate the cell type-specific biophysical properties involved in the generation of complex-spiking output driven by synaptic input through an information-theoretic treatment of their respective transfer functions. Our simulations identify a host of cell type-specific biophysical mechanisms that define the morpho-functional phenotype to shape the cellular transfer function and place these findings in the context of a role for bursting in CA3 recurrent network synchronization dynamics.

## Author summary

The hippocampus is comprised of numerous types of neurons, which constitute the cellular substrate for its rich repertoire of network dynamics. Among these are sharp waves, sequential activations of ensembles of neurons that have been shown to be crucially involved in learning and memory. In the CA3 area of the hippocampus, two types of excitatory cells, thorny and a-thorny neurons, are preferentially active during distinct phases of a sharp wave, suggesting a differential role for these cell types in phenomena such as memory consolidation. Using a strictly data-driven approach, we built biophysically realistic models of both thorny and a-thorny cells and used them to investigate the integrative differences between these two cell types. We found that both neuron classes have the capability of integrating incoming synaptic inputs in a supralinear fashion, although only a-thorny cells respond with bursts of action potentials to spatially and temporally clustered synaptic inputs. Additionally, by using a computational approach based on information theory, we show that, owing to this propensity for bursting, a-thorny cells can encode more information in their spiking output than their thorny counterpart. These results shed new light on the computational capabilities of two types of excitatory neurons and suggest that thorny and a-thorny cells may play distinct roles in the generation of hippocampal network synchronization.

## Introduction

Determining how neurons with distinct morphological and physiological phenotypes differentially process information is a critical step towards understanding how the diversity of cell types in the brain gives rise to the circuit-specific computations that support cognitive function. Among the most critical single-cell computations is the integrative process by which a neuron converts synaptic input to action potential (AP) output (i.e., the neuronal transfer function). Given the breadth of synaptic input patterns each neuron may receive, AP output patterns have a vast degree of heterogeneity, whose biophysical underpinnings are incompletely understood. Specifically, complex spiking or burst firing is a mode of neural output exhibited by numerous cell types throughout the brains of many species spanning a broad range of phylogenetic complexity. This specialized form of information representation and transmission has been postulated to overcome the unreliability of synaptic transmission [1], provide a means of selective routing of information through intrinsic resonance mechanisms [2] and represent a parallel coding dimension relative to single-spike rate coding [3]. Additionally, the biophysical mechanisms of bursting that have been uncovered for mammalian neurons suggest that these events occur in response to conjunctive input to distinct dendritic domains: these manifest biophysically as a dendritic plateau potential upon which a series of high frequency APs can be observed. Moreover, bursting has been shown to be a highly effective means of inducing synaptic plasticity [4–6], which can support information storage and underlie the acquisition of feature selectivity [7–10].

Despite the physiological importance of burst firing, the biophysical mechanisms underlying this activity pattern can be cell type-specific and are incompletely understood for many neuronal types. Towards this end, building data-driven models is an attractive way to test hypotheses about the integrative properties of distinct neuronal cell types that can then be used to simulate how single-cell computations are implemented across divergent morphological and physiological phenotypes. Most previous studies have examined complex spiking behaviour in computational models where the parameters and conductance levels of the biophysical mechanisms were “hand-tuned” [11–15]. While this practice can yield qualitatively similar characteristics between simulations and experiments over a limited range of stimuli, parameterizing models in this way can suffer from getting caught in local minima of the parameter optimization landscape and therefore could potentially display unphysiological behaviours or have a limited dynamic range. To circumvent this pitfall and expedite the often arduous hand tuning process, multi-objective optimization strategies paired with genetic algorithms were developed [16,17]. This innovation has enabled a more robust parameter optimization process that has been widely adopted to generate data-driven biophysical models [18–20].

Here we employ a data-driven approach to develop cell type-specific multi-compartment models of hippocampal CA3 pyramidal neurons based on patch-clamp electrophysiology data and high-resolution morphological reconstructions. We built two distinct populations of individuals capable of recapitulating the firing phenotypes observed in the two principal cell types found in the CA3 region of the hippocampus, thorny regular spiking and a-thorny intrinsically bursting cells [21]. We then demonstrate that the optimized conductance values of the biophysical mechanisms in the models correspond to relative gene expression levels in transcriptomic cell types defined by single-cell RNA sequencing (scRNAseq) data. Specifically, we find concordance between the differentially expressed genes and the cell type-specific ionic conductance values, validating our data-driven approach. We then utilize these biophysically detailed models to investigate the integrative properties of thorny and a-thorny pyramidal cells to gain insight into how their cell type-specific properties influence information transfer capabilities. We find that both principal cell types can integrate synaptic inputs to their dendrites supralinearly, while a-thorny cells display a preference for emitting bursts when stimulated with spatiotemporally correlated synaptic input. The intrinsic tendency of a-thorny cells to preferentially emit complex spikes endows them with a greater capacity to encode information in their firing patterns relative to regular spiking cells. The higher information transmission capacity of a-thorny pyramidal neurons relates to the previously established role for bursts emitted by a-thorny cells as triggers for sharp wave (SW) synchronization events [21]. Viewed within this context, our results shed light on the biophysical mechanisms and integrative processes at the cellular level that promote synchronization dynamics in the CA3 recurrent network.

## Results

### Physiology of CA3 principal cell types

Previously we identified and characterized a novel pyramidal cell type in the CA3 region of the hippocampus, a-thorny principal cells. Together with their thorny cell counterparts, these two principal excitatory cell types comprise the CA3 region of the hippocampus [21]. To further assess the physiological properties of hippocampal CA3 cell types and obtain a broad repertoire of responses to somatic current injection as well as spontaneous synaptic activity, we obtained whole cell recordings in acute slices from CA3 pyramidal neurons in mice. Neurons were sampled in an unbiased manner by blind patching 100–200 μm deep within a 400 μm-thick slice. The intrinsic properties of each cell were characterized by a series of somatic current injections delivered in current clamp mode.

**Fig 1:**
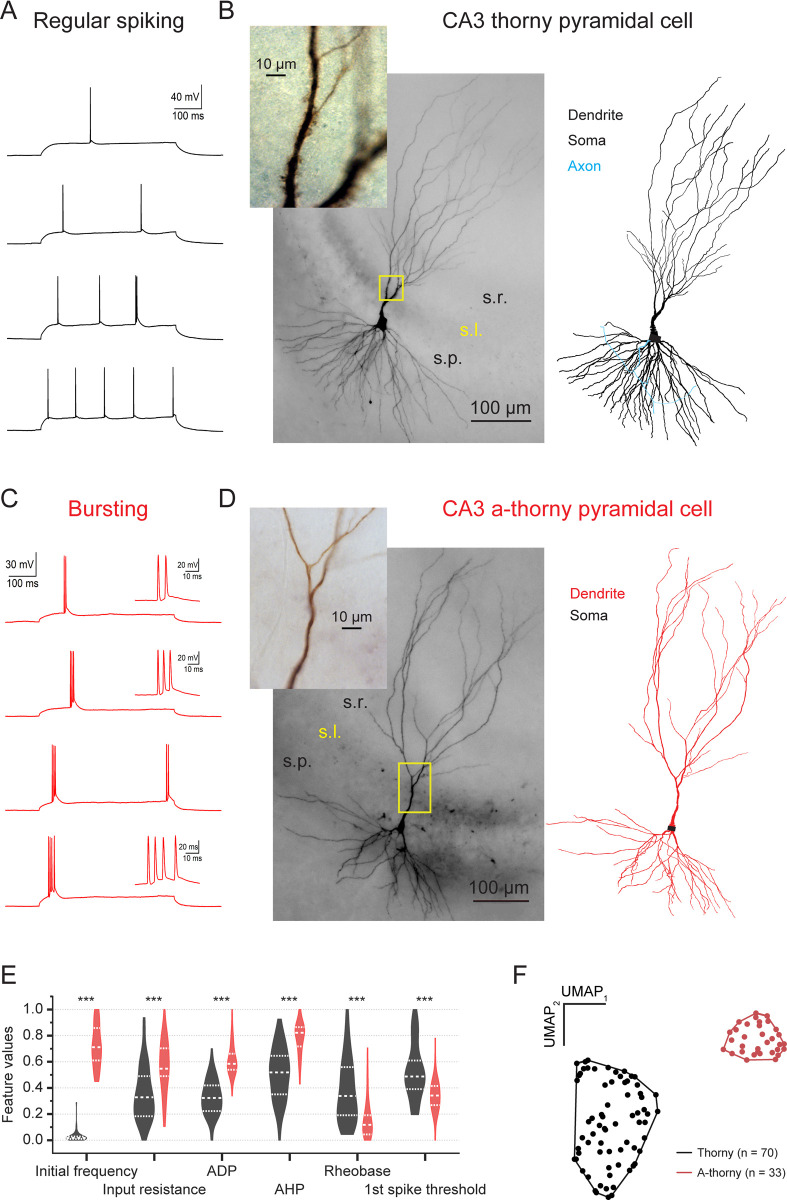
Physiological and morphological phenotypes of CA3 pyramidal neurons. (A) Representative traces from a series of somatic current injections where a regular spiking firing phenotype was observed. (B) Left, biocytin staining of the cell recorded in (A) where a full-scale view of the dendrites and their distribution across the laminar structure of the CA3 region is shown. Inset, high magnification view of the proximal apical dendrite where prominent complex spiny structures known as thorny excrescences can be observed. Right, 3D morphological reconstruction of the biocytin-stained cell. (C) Representative traces from a series of somatic current injections where a bursting phenotype was observed. (D) Left, biocytin staining of the cell recorded in (C) where a full-scale view of the dendrites and their distribution across the laminar structure of the CA3 region is shown. Inset, high magnification view of the proximal apical dendrite where a clear lack of complex spines (a-thorny) is observed. Right, 3D morphological reconstruction of the biocytin-stained cell. (E) Summary of physiological features (normalized in the range of variability of each feature over both cell populations) highlighting several key physiological differences across the populations of regular spiking and bursting cells. (F) Clustering of physiological features for all recorded cells clearly shows two main cell classes that correspond to regular spiking (thorny) cells and intrinsically bursting (a-thorny) cells.

Representative cell morphologies and firing patterns for a range of somatic current injections of each cell type are shown in Fig 1. As shown in panels A and C, several key physiological differences exist between CA3 principal cells: most notable is their firing pattern near rheobase, where thorny cells exhibit a regular spiking phenotype, while a-thorny cells are characterized by an intrinsically bursting phenotype. Importantly, the two distinct firing phenotypes tightly correlate with key morphological differences between cell types, as illustrated in Fig 1B and 1D. Specifically, regular spiking cells display prominent thorny excrescences, the complex spiny structures where mossy fiber axons from the dentate gyrus form synapses with mossy cells of the hilus and CA3 pyramidal neurons. On the contrary, intrinsically bursting cells lack these postsynaptic structures and receive little to no input from dentate granule cells [21]. The results of a thorough electrophysiological characterization of the two cell types are reported in S1 Table: additionally, Fig 1E shows violin plots of six electrophysiological features (normalized to their range of variation) that were significantly different between the two cell types. In Fig 1F these same data are plotted using the UMAP algorithm [22] for dimensionality reduction, which clearly shows how thorny and a-thorny cells segregate in two distinct clusters of individuals, indicating that these cells constitute two separate populations of principal neurons in the CA3 region of the hippocampus.

### Biophysical models of CA3 pyramidal cell types

CA3 thorny and a-thorny cells are characterized by radically different morphological and electrophysiological features and play crucially distinct roles in CA3 network dynamics [21,23]. To capture the defining features of these phenotypes in detailed biophysical models of these cells, we employed a multi-objective optimization framework paired with a genetic algorithm using BluePyOpt [17]. This open-source Python package enabled us to explore a wide range of parameter combinations and ultimately tune the parameters of the models such that the behavior of the model closely matched the features extracted from somatic current injection experiments. We utilized an array of ionic mechanisms with distributions appropriate for CA3 pyramidal neurons [20], with two important distinctions aimed at better capturing the bursting phenotype characteristic of the a-thorny cell type. First, we included a persistent sodium current located perisomatically [24], and secondly we chose to optimize the parameters in the model that regulate intracellular calcium dynamics—namely the time constant of calcium buffering and the availability of free calcium inside the cell, yielding a total number of 24 free parameters. The main strength of the multi-objective optimization strategy implemented by BluePyOpt is that it produces a family of solutions, called individuals, rather than a single optimal value. Individual solutions to the optimization comprise a final population of models that satisfy the constraints imposed by the target features and occupy the pareto-optimal frontier [16,25]. Since the error on each feature is measured in units of standard deviations from the experimental mean, this strategy leads to a population of individuals whose intrinsic variability recapitulates that observed in acute brain slice experiments.

**Fig 2:**
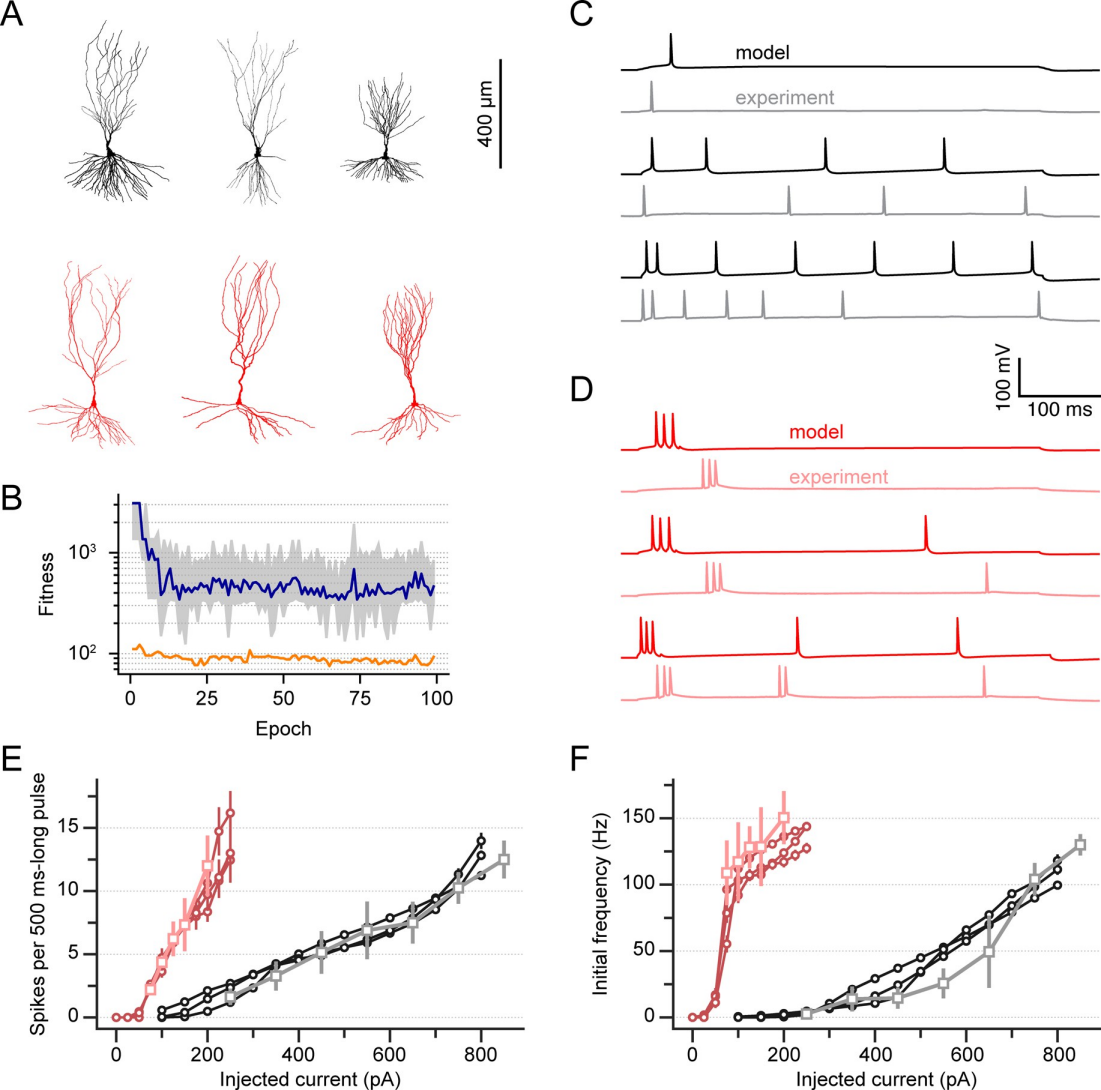
Biophysically realistic neuron models recapitulate the distinct electrophysiological phenotypes of CA3 pyramidal cells. (A) Detailed thorny (top, black) and a-thorny (bottom, red) cell morphologies used in this study. (B) Typical evolution of the population fitness as a function of optimization epoch for an a-thorny cell model: the grey shaded area represents the range of the 25th and 75th percentile, the blue trace is the median population fitness, and the orange trace is the fitness of the best individual at each epoch. Similar results were observed for the optimization of thorny cell models. (C) Representative somatic voltage traces for a thorny regular spiking cell in response to increasing levels of constant injected current in the model and in the experiment (black and grey traces, respectively). (D) Same as (C), but for the a-thorny intrinsic bursting cell type (red and pink traces are model and experiment Vm, respectively). In both (C) and (D), notice how the models are capable of reproducing the firing phenotype both at the onset of the stimulus and for the overall duration of the stimulation. (E) Static input-output relationships (f-I curves) in the models and in the experiments, computed as the total number of spikes emitted during stimulation divided by the length of the stimulus, for thorny and a-thorny cells (black and red traces, respectively). Markers and error bars indicate mean and SEM, respectively. (F) Initial firing frequency as a function of the injected current in the models and in the experiments. Color code is the same as in (E).

As a component of our optimization workflow, we utilized three reconstructed morphologies per physiological cell type (Fig 2A) and performed several optimization runs for each morphology. Typically, each run was composed of 128 or 144 individuals and evolved for 100 to 150 generations at which point the reduction in error across subsequent generation plateaus (Fig 2B). From the final set of solutions of the multi-objective optimization, we selected individuals whose error for each feature was below 6 standard deviations from the mean, ensuring that the behavior of each individual model matched that of the cells used as targets for the optimization. This led to a grand total of 180 and 172 individuals that met the inclusion criterion for thorny and a-thorny cells, respectively. These individuals were subdivided across the three morphologies used in the optimization for each cell type. As shown in Fig 2C and 2D, the membrane voltage (V_m_) traces obtained with the models in response to the injection of constant steps of current of increasing amplitude (black and red traces for thorny and a-thorny cells, respectively) are qualitatively very similar to the corresponding experimental recordings (gray and pink traces for thorny and a-thorny cells, respectively), for both cell types. Importantly, the thorny cell models (Fig 2C) are typically capable of reproducing both the overall regular firing phenotype observed in the experiments and the high-frequency AP doublet observed in most thorny cells at the onset of stimulation for high enough values of injected current. The a-thorny cell models, on the other hand, display the characteristic high-frequency bursts observed in the patch-clamp experiments (Fig 2D), with a slight increase in overall firing rate as the magnitude of the injected current is increased, in agreement with our data.

To more precisely assess the quality of the model responses, we computed the static input-output relationships (f-I curves) for all individuals that met the quality criterion indicated previously by injecting 500 ms-long direct current injection steps, similarly to what was done in the patch-clamp experiments. We then measured the overall firing rate as the number of spikes during the 500 ms-long interval of current injection and the initial firing rate as the inverse of the inter-spike interval of the first 2 spikes in the train. Fig 2E and 2F shows the f-I curves of the models averaged based on the morphology (black and red circular markers and error bars for thorny and a-thorny cells, respectively). The agreement with the experimentally measured f-I curves (gray and pink square markers and error bars) is high for both the overall firing rate (Fig 2E) and for the initial firing rate (Fig 2F). Additionally, the models correctly capture the difference in terms of rheobase that can be observed experimentally (grand-average rheobase values for all the individuals obtained with the optimization: 200 ± 4.2 pA vs. 69 ± 1.1 pA for thorny and a-thorny cells, respectively, *p* < 10^−10^, Student t-test). Together these data indicate that our data-driven approach is a robust method to produce models that faithfully recapitulate the biological phenotype of the individual cell types.

### Comparison between model parameter distributions and scRNAseq data of CA3 excitatory neurons

**Fig 3:**
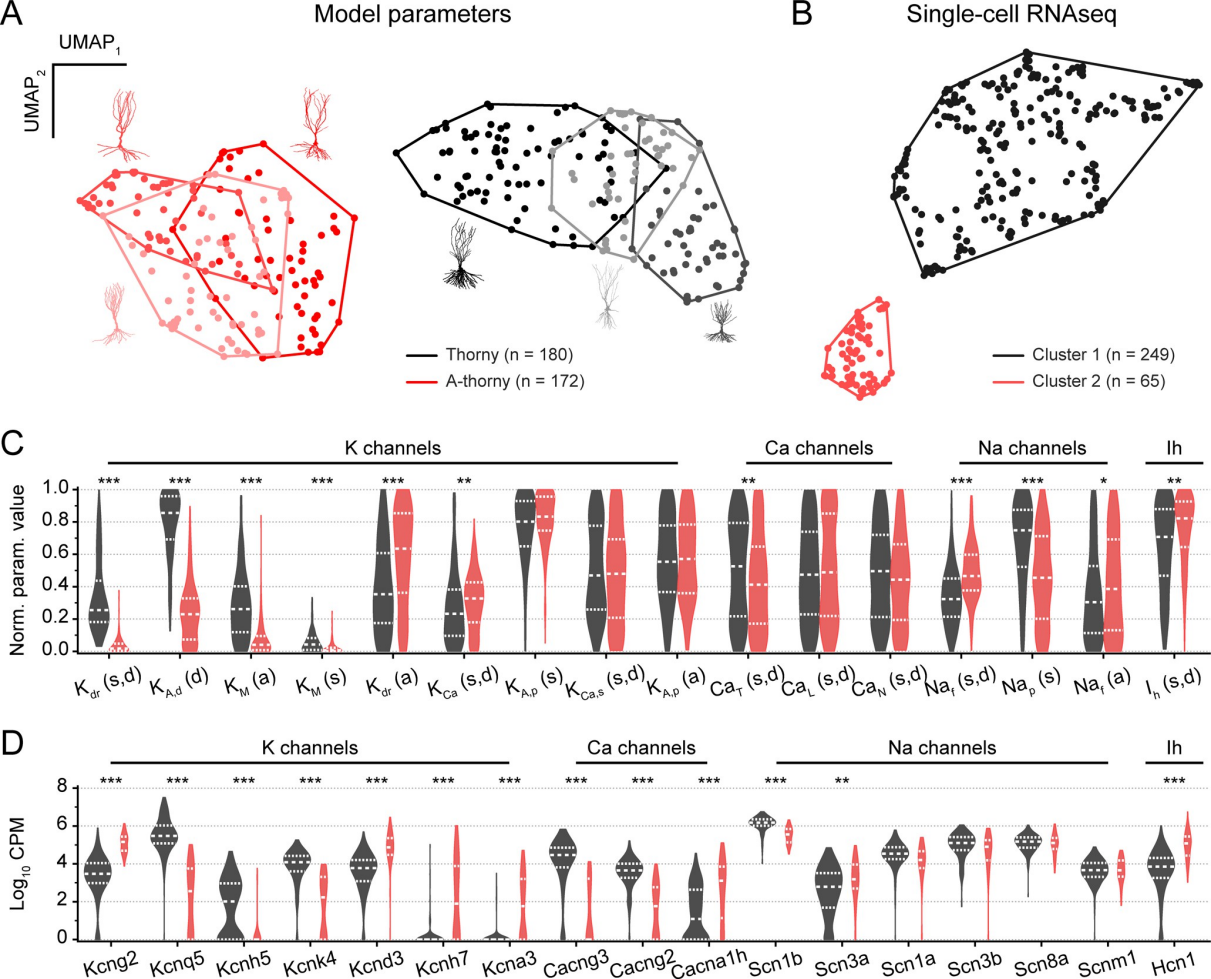
Distributions of model parameters mirror differentially expressed ion channel genes. (A) Bidimensional representation of parameter values transformed using UMAP: each dot represents one individual, and closed lines indicate the convex hulls associated with all the individuals obtained with a given morphology (color-coded accordingly to both the convex hull and the points contained in it). (B) UMAP projection and clustering of CA3 excitatory neurons based on scRNAseq data. Using the Leiden clustering algorithm with a resolution of 0.65 delineated the primary division in the CA3 principal neuron population. Note that CA3 principal cells are primarily composed of a larger population of cells (cluster 1, black) and a second minority population (cluster 2, red). (C) Violin plots of the distributions of maximal conductance values for four different classes of ion channels (potassium, calcium, sodium and hyperpolarization-activated) for the model cells included in the analysis, normalized over the range of allowed variability of each parameter as reported in S2 Table (black and red indicate thorny and a-thorny cells, respectively). Dashed lines indicate the median of the population, while the upper and lower dotted lines represent the 25th and 75th percentile of the distributions. Most parameter distributions were significantly different between the two cell-types (non-parametric Kolmogorov-Smirnov test: * p < 0.05, ** p < 0.01, *** p < 0.001). For the remaining parameters see S1 Fig. (D) Expression levels for cells belonging to cluster 1 (black) or cluster 2 (red) for analogous classes of ion channel genes as shown in (C). Note that expression levels for most Na channel genes were not significantly different while Ca and K channel genes were significantly differentially expressed between the two clusters (non-parametric Kolmogorov-Smirnov test: * p < 0.05, ** p < 0.01, *** p < 0.001).

Given that the evolutionary optimization we employed produces a set of individuals that meet the optimization constraints to varying degrees, we investigated the differences in the sets of parameter values between thorny and a-thorny cells and compared them with scRNAseq data. The aim of this analysis is twofold: first, it provides a quantification of between- and within-cell type heterogeneity and of the influence of morphology on firing behavior. Secondly, it elucidates which parameters account for the increased propensity of a-thorny cells to generate bursts of APs. We started by mapping the parameters of the accepted individuals from the original 24-dimensional space to a 2-dimensional one by using the UMAP algorithm [22]: as shown in Fig 3A, a-thorny individuals (red dots) clustered more closely, and no major effect of the different morphologies was observed. On the other hand, thorny cells (black dots) appeared as a continuum, with the cell morphology playing a more prominent role in how individuals clustered. Interestingly, we found a very similar picture when using UMAP to visualize scRNAseq data from CA3 excitatory neurons [26], as shown in Fig 3B. By using all genes and the Leiden clustering algorithm [27], we obtained a major subdivision in the cell population clearly separating CA3 principal cells in two main clusters: a more numerous and more dispersed one (black dots) and a tightly clustered smaller group (red dots). These results are consistent with previous evidence for two main morpho-functional cell types corresponding to thorny and a-thorny cells where the a-thorny neurons are a minority population.

The differences in parameter distributions between the two cell types are shown in Fig 3C, which displays violin plots of the parameters normalized to their range of variability during the optimization process and grouped according to ion channel species. The most prominent differences can be observed in potassium currents, which are down-regulated in a-thorny cells. Interestingly, among calcium currents only the T-type one is down-regulated in a-thorny cells, while the other two calcium conductances are non-significantly different between the two cell types (two-sample Kolmogorov-Smirnov test). On the other hand, the main (fast) sodium current, responsible for AP initiation, is up-regulated in a-thorny cells, indicating an increased excitability in this cell type. Calcium dynamics are also significantly different between the two cell types (see S1 Fig for parameter distributions not shown in Fig 3C): the time constant of calcium buffering is significantly larger in a-thorny cells, while the availability of free calcium is lower. This interplay of slow and fast variables in the a-thorny cell models is at the basis of their bursting capabilities [24,28]. This finding suggests that two types of bursting might be present in CA3 principal cells: on the one hand, a somatically generated bursting that relies on the relative contribution of sodium and potassium current can be observed in response to the somatic injection of constant steps of current. On the other hand, dendritic plateau bursting can be elicited by ongoing synaptic activity, as will be shown in the following sections.

To further validate the optimization results, we performed differential expression analysis of the scRNAseq data between the cell clusters shown in Fig 3B. While we observed many differentially expressed genes at the significance thresholds that we implemented, we chose to focus specifically on ion channel genes to compare how differential expression of ion channels relates to the difference in conductance values in our cell type-specific models found by our optimization algorithm. We found several important consistencies between the conductance values that impart the cell type-specific firing phenotype in the models we developed and the expression levels for ion channel genes that correspond to those biophysical mechanisms. Once again, we focused on the three major conductance types present in our models, namely sodium, potassium, and calcium. Each of these three classes of conductance types has several mechanisms in the model corresponding to distinct channel sub-types that in turn are related to several channel subunit genes. To relate the conductance values in the models to the differences in expression of ion channel genes between the two main clusters (see S2 Fig), we examined the expression level for key ion channel genes that correspond to biophysical mechanisms in our models (Fig 3D). Analogously to what we observed for the distributions of model parameters, we found significant differences in the expression levels of potassium and calcium channels, while the expression levels of sodium channels were largely unchanged between the two cell groups. Together these data provide independent biological validation of the data-driven approach we have taken, where the critical conductance differences resulting in distinct physiological phenotypes are found by the optimization algorithm and at the same time correspond to actual gene expression differences between transcriptomic cell types, providing an example of multi-modal integration between physiology, morphology, biophysical simulation, and scRNAseq data.

### Quantification of dendritic input resistance in active cell models

**Fig 4:**
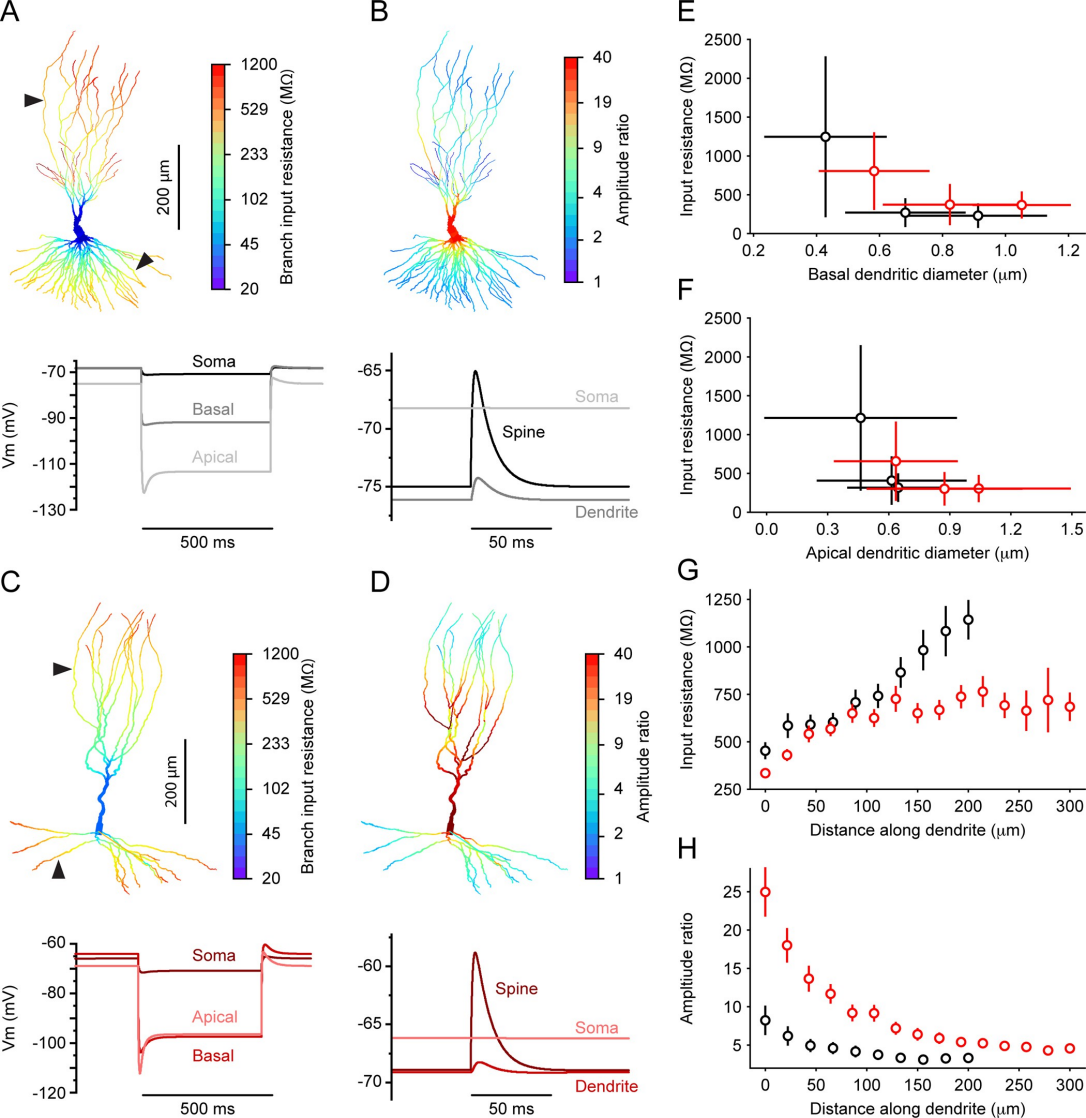
Distinct dendritic properties of cell type specific models. (A) Representative distribution of branch input resistance (R_in_) values in one of the thorny morphologies considered in this study. Top: warmer colors indicate higher values of R_in_ and black arrowheads indicate the recording locations for the sample voltage traces shown at the bottom of panels (A) and (B). Bottom: example voltage traces in response to the injection of a 500 ms-long hyperpolarizing current in the soma and in one compartment of the apical and basal dendrites. (B) Representative distribution of amplitude ratio values in the same individual shown in (A). Top: warmer colors indicate higher values of amplitude ratio and have an inverse relationship to R_in_. Bottom: example voltage traces recorded in the spine head, dendrite shaft and soma in response to the injection in the spine head of an EPSP-shaped current whose amplitude was dynamically adjusted to cause a Vm deflection in the spine of approximately 10 mV. (C, D) Same as (A, B), but for a representative individual of the a-thorny cell type. Notice how the highest values of R_in_ in the thorny cells are located in the apical obliques while in the a-thorny cells R_in_ generally increases with distance from the soma. (E, F) R_in_ as a function of basal and apical dendrite diameter for the three morphologies shown in Fig 2 for each of the cell types. Dots and bars indicate mean and standard deviation, respectively. (G) R_in_ as a function of the distance along distal apical dendrites (i.e., unbranched stretches of dendrites comprised between a bifurcation and the tip of the dendrite) for thorny and a-thorny cells (black and red markers, respectively). Dots and bars indicate mean and SEM, respectively, computed over the morphologies shown in Fig 2. (H) Same as (G), but for the amplitude ratio of synaptic inputs.

We then sought to investigate the differences in the biophysical and functional properties between the two principal cell types found in CA3 utilizing the tuned biophysical models. First, we analyzed the differences in input resistance (R_in_) values throughout the dendritic tree between thorny regular spiking and a-thorny bursting cells. To this end, we injected 500 ms-long hyperpolarizing steps of current in each compartment of the dendritic tree to measure its R_in_: as reported previously [29], we found that branch R_in_ is inversely correlated with the dendrite diameter and thus increases with the distance of the dendritic branch from the cell body (Fig 4A and 4C). Fig 4 summarizes the results of these simulations for the three cell morphologies used in the optimization procedure, where we separated results based on whether the branch was located on the basal or apical dendrite. As shown in Fig 4E, at the population level a-thorny cells tend to have slightly thicker basal dendrites with higher R_in_, while that measured on the apical dendrites is comparable between the two cell types (Fig 4F). To compare thorny and a-thorny cells more precisely, we identified terminal apical branches in the a-thorny cell morphologies and apical oblique branches in the thorny cell morphologies: the latter are a prominent feature of the thorny cell type, while being completely absent in the a-thorny morphologies. The reason for this choice is twofold: first, it allows us to compare thorny and a-thorny cells by looking at terminal unbranched stretches of dendrites, and secondly it makes it possible to compare thorny cells with results present in the literature about CA1 pyramidal cells [29]. The R_in_ of these branches as a function of the distance from the originating branch point is plotted in Fig 4G: the dependence on the distance along the dendrite for thorny cells is reminiscent of what has been observed previously for CA1 pyramidal cells [29], while the higher R_in_ values observed in the terminal branches of a-thorny cells suggest that these dendritic branches might be particularly suited to input compartmentalization and the generation of nonlinearities by active conductances. We computed dendritic R_in_ using a model of active cells in which sodium channel conductances were removed, simulating bath application of TTX. This was done to have the same experimental condition as the patch-clamp experiments on the amplitude ratio reported in the next section.

### Determination of amplitude ratio for synaptic input onto dendritic spines

Given the differences in dendritic input resistance observed in the two cell type models, we investigated how synaptic inputs localised on dendritic spines are spatially and electrically segregated from the rest of the dendritic tree. Consistent with previous studies [29], we modelled spines as two cylindrical compartments representing head and neck (head length and diameter: 0.5 μm, neck length and diameter: 1.58 μm and 0.077 μm, respectively, unless noted otherwise). Spines had the same axial resistance as their parent dendritic branch and in these simulations contained only passive channels, whose properties matched those of the dendritic branch where the spines were connected. To compute the amplitude ratio (AR) between spines and dendritic tree, we injected an EPSP-shaped current into the spine whose maximum amplitude was dynamically adjusted to elicit a V_m_ deviation in the spine of approximately 20 mV. The AR was then defined as the ratio of the EPSP measured in the spine to that elicited in the dendrite (different values were also tested and did not lead to significant changes in the computed values of AR). Fig 4B and 4D shows the results of these simulations, for one representative morphology for each cell type, where the AR is correlated with the branch R_in_ and with the diameter of the dendrite. By focusing on the subset of apical dendritic branches discussed previously for the R_in_ measurements, we found that the amplitude ratio drops with the distance along the oblique dendrites and the terminal branches for thorny cells and a-thorny cells, respectively. However, there is a marked difference in the values of AR for the two cell types: those for the thorny cells are significantly smaller than those observed in CA1 pyramidal cells (which can be at least partially explained by the fact that the experiments of [29] were carried out in rat cells), while the AR for a-thorny neurons is about twice as much as that of CA1 pyramidal cells and almost one order of magnitude larger (at least close to the branch points) than the AR in thorny cells. In the same branches, a-thorny cells display larger dendrite diameters and smaller R_in_, which, together with the active ionic conductances, account for the difference in AR values. This and the higher R_in_ observed in these same dendrites lend credit to the idea that a-thorny cells might be better suited than thorny cells at compartmentalizing the synaptic inputs impinging on their dendritic trees.

### Cell type-specific synaptic cooperativity and nonlinear synaptic integration

**Fig 5:**
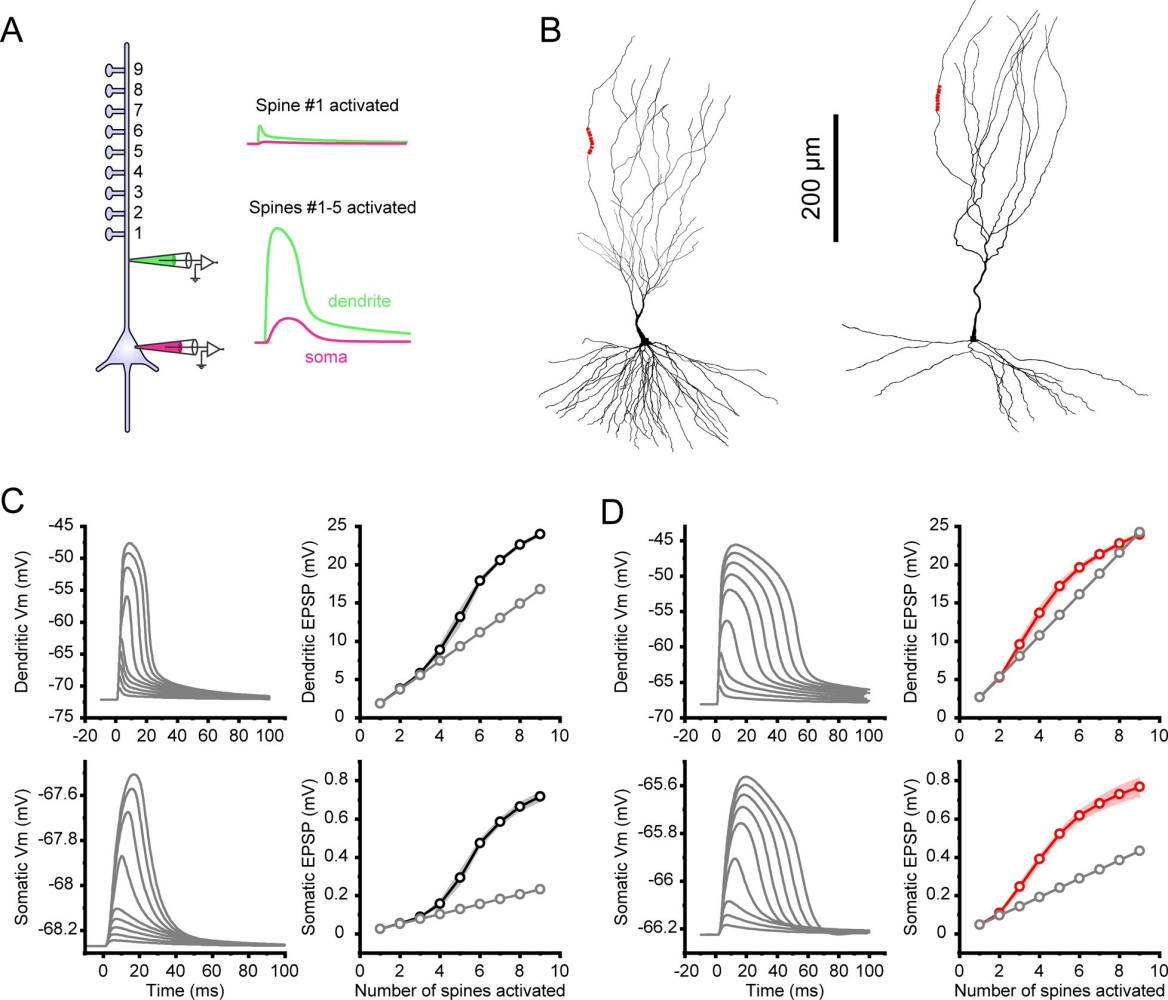
Cooperativity and nonlinear amplification of synaptic input in cell type specific models. (A) Schematic of the experimental setup: 9 spines were placed on an apical branch and stimulated sequentially by the arrival of presynaptic spikes with a time interval of 0.3 ms. Each spine contained AMPA and NMDA synapses, whose parameters were adjusted to lead to a voltage deflection at the spine head of approximately 20 mV. The spine EPSP then propagated to the dendritic trunk and soma (example voltage traces shown on the right) when one or multiple spines were activated. (B) Location of the spines (red dots) on the apical branch of a thorny (left) and an a-thorny cell (right). (C) Vm traces recorded in the dendritic trunk (top left) and at the soma (bottom left) and corresponding EPSP peak amplitudes (right, black markers) in response to increasing numbers of presynaptic inputs in the thorny cell shown in (B, left). Grey markers indicate the linear prediction obtained by multiplying the amplitude of the EPSP in response to a single input by the number of presynaptic inputs. (D) Same as (C) but for the a-thorny cell shown in (B, right).

Following the results demonstrating that dendritic spines in both thorny and a-thorny cells provide cell type-specific compartmentalization of synaptic inputs, we included AMPA and NMDA receptors in the spines and simulated the arrival of clustered synaptic inputs on up to nine neighbouring spines on an apical dendritic branch, as shown schematically in Fig 5A, and for the two morphologies considered in Fig 5B. We tuned the weights of the AMPA and NMDA synapses to have a V_m_ deflection in the spine head of approximately 20 mV, with a long tail due to the NMDA component (NMDA decay time constant was set to 50 and 100 ms, for thorny and a-thorny cells, respectively, to match experimental data, see S3 Fig). The other synapse parameters for AMPA and NMDA were the same for both cell types (see Materials and Methods). When only one synaptic input was activated, the cell response was similar to what we observed in the previous set of experiments with the direct injection of EPSP-shaped currents in both the spine head and dendritic shaft. Also, we obtained a marked reduction between the EPSP_spine_ and the EPSP_dend_. For the representative a-thorny cell shown in Fig 5B (right morphology), this led to an AR value of approximately 7.3, while the thorny cell shown in Fig 5B (left morphology) had a slightly higher value of AR of approximately 9.5, indicating a higher compartmentalization of spine inputs in the more distal branches of thorny cells compared to their a-thorny counterparts.

We then increased the number of concomitant synaptic inputs arriving on neighbouring spines from 1 to 9 and measured the V_m_ deflection in the dendrite and at the soma. The distance between spines was set to 5 μm, although our results are consistent across other values of inter-spine distance. The time evolution of the V_m_ for one representative individual is shown in Fig 5 (on the left of panels C and D for the thorny and a-thorny cell, respectively). For the a-thorny individual shown in Fig 5D, the arrival of four concomitant presynaptic inputs is sufficient to elicit a supralinear response, while in the case of the thorny cell (Fig 5C), the number of required presynaptic inputs is equal to five. We remark however that these numbers depend on the AMPA and NMDA weights and are therefore not indicative of the actual number of required inputs but rather serve to illustrate the mechanism at the base of the supralinear summation of inputs likely to be observed in these cells, in agreement with what has been reported previously for CA1 pyramidal cells [29,30]. We then considered a population of individuals resulting from one optimization run to plot the mean ± SEM of dendritic and somatic EPSPs as a function of the number of inputs (on the right of panels C and D for the thorny and a-thorny cell, respectively). The supralinearity of the response is especially evident in the somatic EPSPs, which display values in line with the experimentally observed ones [21]. These results indicate that, when NMDA receptors are present, clustered synaptic inputs on the apical dendrite can generate supralinear responses in both cell types. However, our results suggest that, due to the unique morphological differences that determine the dendritic branch input resistance profile, a-thorny pyramidal cells can reach the supralinear regime with proportionally fewer synaptic inputs. This property can endow this cell types with a lower threshold to reach the supralinear regime and/or a longer integration time window for the same level of synaptic drive.

**Fig 6:**
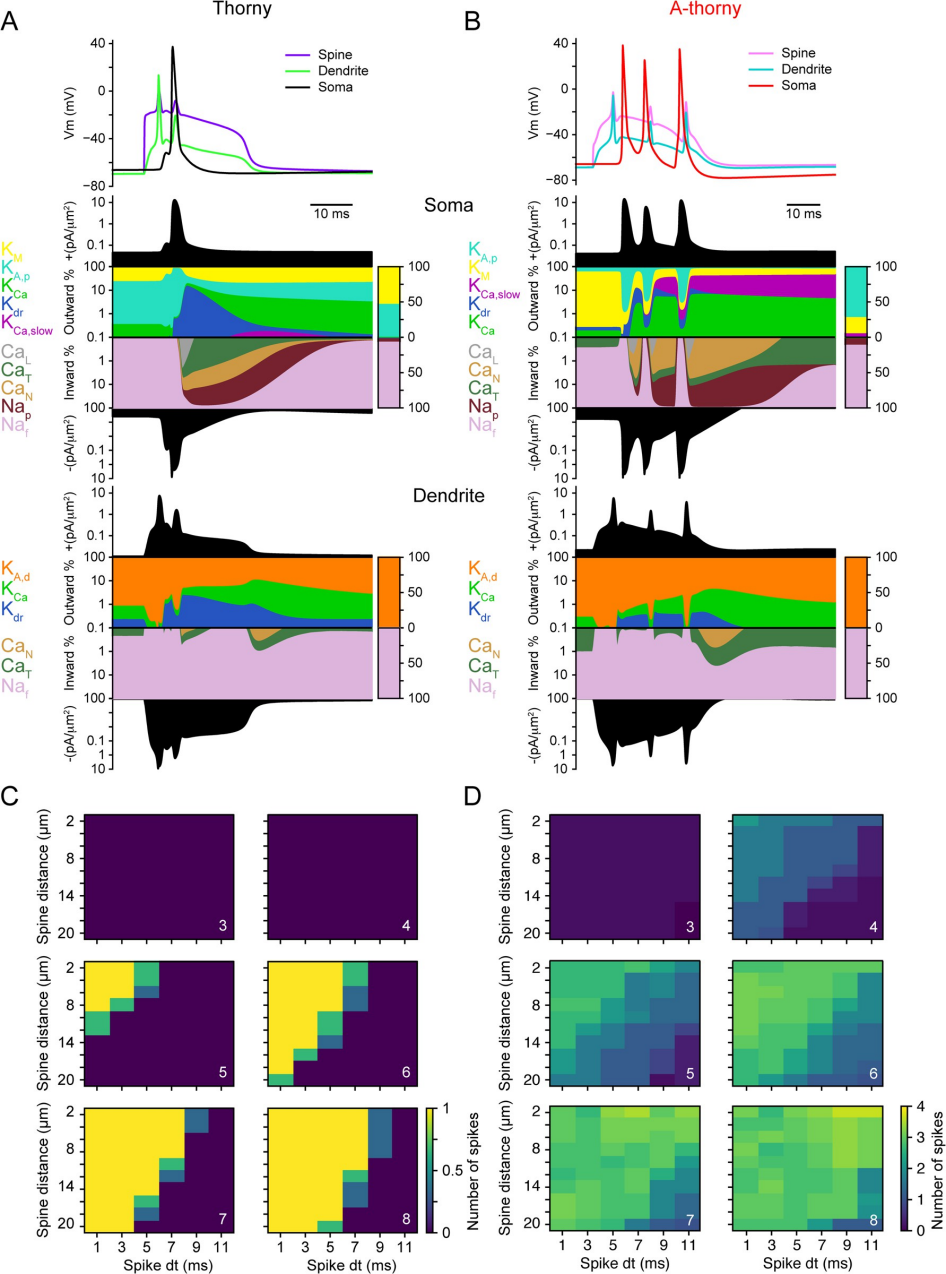
Distinct responses of thorny and a-thorny cells to clustered dendritic inputs. (A) Top, simulated Vm dynamics in a thorny cell in response to the concomitant arrival of 6 presynaptic inputs. The spine and dendrite Vm clearly show the dendritic plateau that lies at the origin of the AP that is then propagated at the soma. Middle and bottom, visualizations of the dynamics of inward and outward ionic currents (i.e., “currentscapes”) respectively at the soma and at the dendritic location where the stimulated spine is connected. In each currentscape, the filled black areas represent the total amount of outward and inward current densities (top and bottom, respectively), while the middle panels quantify the relative contribution of each ionic current to the total. The logarithmic scale highlights the dynamics of those currents that contribute little to the total. The color bars on the right summarize the percent contribution of each current type to the total inward and outward current densities, and are shown on a linear scale. (B) Same as (A), but for an a-thorny cell: the dendritic origin of the somatic burst is evident and accounts for the steep onset of the first AP in the burst, while the subsequent APs are the result of the interplay between somatic and dendritic ionic currents. Note the different relative contribution of calcium-activated potassium currents to the total outward current density measured at the soma. (C) Mean number of spikes emitted by a population of thorny cells in response to a varying number of synaptic inputs (indicated in white in each panel) arriving at different frequencies (on the x-axis) on spines that are located at different distances (on the y-axis). (D) Same as (C), but for a population of a-thorny cells.

To complete these simulations, we set the sodium conductances to the values provided by the optimization procedure and stimulated the models while varying the number of incoming synaptic inputs. The results of this final set of experiments are shown in Fig 6: example V_m_ traces recorded in the soma, spine head and dendritic trunk are shown in panels A and B, for representative thorny and a-thorny cells, respectively. The concomitant arrival of a sufficiently high number of synaptic inputs causes a pronounced V_m_ deflection in the spine head, which propagates towards the soma as a dendritic plateau [30,31]. To dissect the precise contribution of each ionic current to the somatic and dendritic Vm time course, we plotted the dynamics of inward and outward currents by means of a “currentscape” [32], which allow visualizing the relative contribution of each ionic current (and its time course) to the total inward and outward current densities. Notably, thorny and a-thorny cells differ in the amount of dendritic T-type calcium currents (dark green band in the bottom panels of Fig 6A and 6B), in the relative contribution of several potassium channels to the total outward somatic current, and in the amount of persistent sodium currents, which are larger in a-thorny than in thorny cells (middle panels of Fig 6A and 6B). These results are in line with previous works that have identified the interplay between potassium and persistent sodium currents as the key mechanism at the basis of neuronal bursting [24]. Currentscapes also make evident how the somatic depolarization induced by dendritic currents activates perisomatic currents, which in turn lead to a rapid burst of APs in a-thorny cells, while in thorny cells the AP output is limited to one. This interaction between dendritic and somatic compartments is evident in the relative timing of somatic APs and dendritic “spikelets”: the first dendritic spikelet precedes the first somatic AP, indicating propagation from the dendritic domain towards the soma, whereas the subsequent spikelets follow the corresponding somatic AP, indicating dendritic backpropagation of somatically generated APs. In order to test the robustness of this phenomenon, we performed the same experiment in the population of individuals employed for Fig 5C and 5D, while varying the (presynaptic) inter-spike interval (ISI) and the distance among spines: the results are shown in Fig 6C and 6D, where one can clearly see that the average number of spikes in a burst for a-thorny cells appears as a continuum that depends both on ISI and spine distance, whereas the response of thorny cells is much more “binary”.

### A-thorny cells transmit more information in response to clustered synaptic input

**Fig 7:**
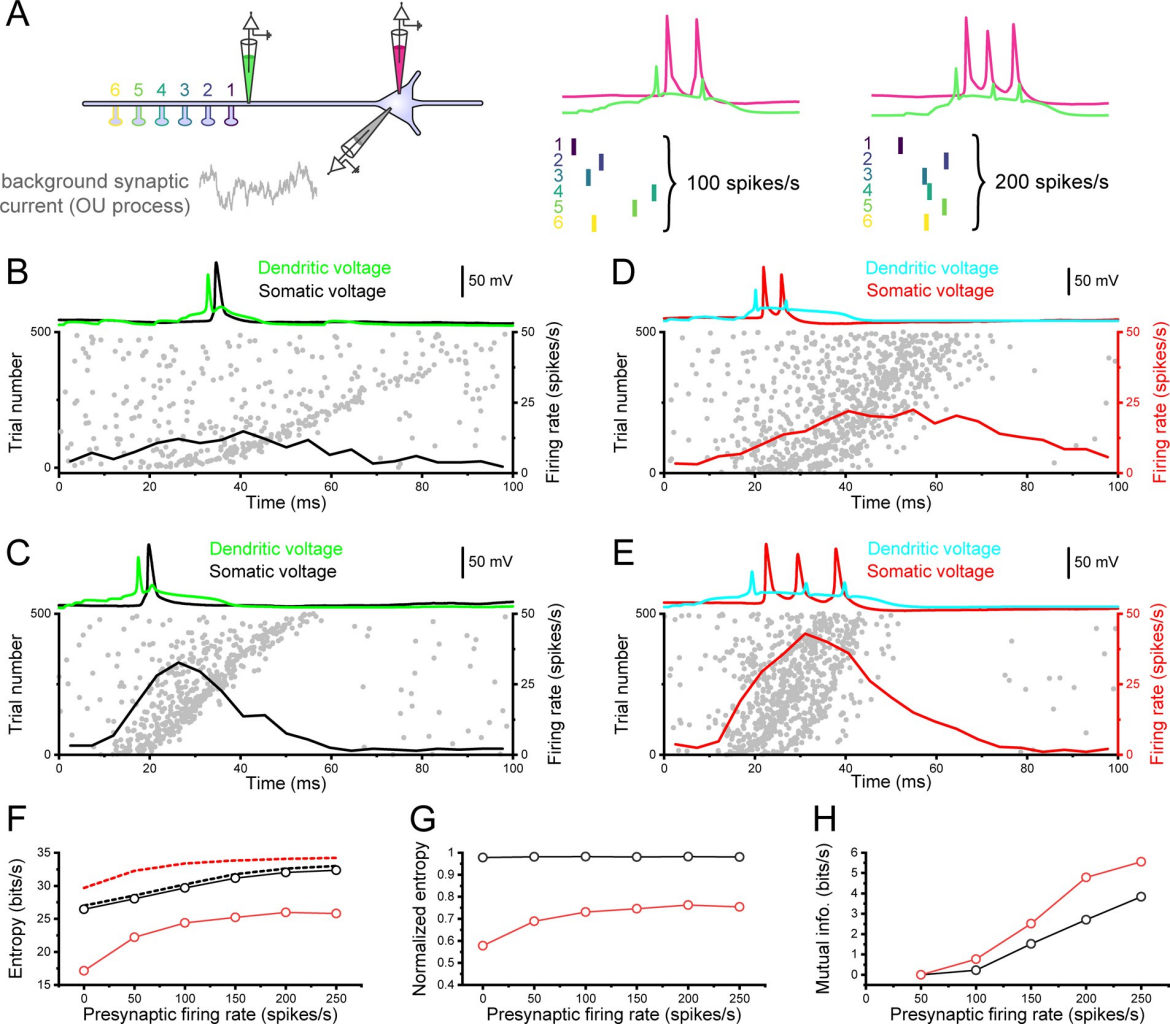
Firing patterns of a-thorny cells encode more information about the statistics of presynaptic inputs. (A) Schematic of the experimental design: left, six spines are placed on the apical dendrite of a cell that is simultaneously stimulated by the somatic injection of an OU process mimicking the arrival of a large number of asynchronous inputs impinging on the dendritic tree. The magenta and green pipettes represent the somatic and dendritic recording locations, respectively. Right, example somatic and dendritic Vm traces in response to a volley of presynaptic spikes arriving at 100 or 200 spikes/s. (B) Spiking dynamics of a thorny cell in response to the injection of both a noisy current at the soma, mimicking the arrival of a large number of presynaptic inputs, and of 6 additional dendritic inputs whose inter-stimulus intervals are distributed according to a Poisson distribution at a rate of 100 spike/s. Each dot in the raster plot represents one postsynaptic AP and each row shows the APs emitted by the cell in response to the arrival of one presynaptic burst, with time being aligned to the arrival of the first presynaptic AP in the burst. Trials are ordered according to the duration of the presynaptic bursts, with shorter bursts having lower trial numbers. The top trace shows the dendritic and somatic Vm in response to the arrival of one presynaptic burst. The black trace represents the firing rate of the thorny cells computed over all trials. (C) Same as (B), but for presynaptic bursts arriving at a mean firing rate of 200 spike/s. (D, E) Same as (B, C), but for an a-thorny cell. Notice how this cell type preferentially responds with bursts as the presynaptic firing rate increases. (F) Entropy of the post-synaptic spike train for thorny and a-thorny cells (black and red markers, respectively) as a function of the presynaptic intra-burst firing rate. The black and red dashed lines represent the theoretically maximal entropy achievable by a point process at the same firing rate as that of the corresponding cell type. (G) Same data as shown in (F) but normalized to the maximal achievable entropy. (H) Mutual information between the postsynaptic firing times and the presynaptic burst times (i.e., the time of the first AP in the presynaptic burst), as a function of the presynaptic intra-burst firing rate.

Given the cell type-specific integrative properties we demonstrated above, we wondered whether the amount of information transferred to the output spike train might be different between the two cell types. To test this hypothesis, we employed the experimental protocol shown schematically in Fig 7A where we injected in the soma of each cell a current modelled by an Ornstein-Uhlenbeck process [33]. This current is intended to mimic the background synaptic activity experienced by a cell in vivo [34,35]. We then placed six spines on the dendritic tree of each cell (in the same location as shown in Fig 5B) and activated them with brief bursts of presynaptic APs, whose arrival times (within each burst) were generated by a Poisson process. The burst times were also Poisson-distributed with a mean burst rate of 2 Hz. We increased the average firing rate of the within-burst Poisson process from 50 to 250 Hz while keeping the overall firing rate of the cell approximately constant (around 5 spike/s). As shown in Fig 7B and 7D, increasing the within-burst presynaptic firing rate from 100 to 200 Hz in a thorny cell only reduces the delay in response onset, thus making the peri-stimulus time histogram computed over all trials (black traces in the bottom part of panels B and D) steeper. While this reduction in response onset is present also in the a-thorny cell (Fig 7C and 7E), higher presynaptic firing rates also cause an increase in the proportion of bursts with 3 or more APs.

To quantitatively assess whether the different firing patterns produced by the two cell types have an effect on the amount of information they are capable of transmitting, we computed both the entropy of the output spike train and the mutual information between the timing of presynaptic burst onset (i.e., the time of the first AP in each burst) and the output APs using the context-tree weighting algorithm [36,37]. We found that the entropy of the output spike trains increases with the presynaptic firing rate in both cell types (Fig 7F): however, to better discern the contribution of the change in spiking output structure from that of the modest increase in output firing rate, we normalized the values of entropy to those that would be obtained with a Poisson process at the same overall firing rate. Indeed, Poisson processes produce spike trains with maximal entropy that increases with firing rate: the theoretical value of entropy (measured in bits s^-1^) is given by $r\frac{{log}_{2}(e)}{r\Delta t}$ where *r* is the firing rate of the process, Δ*t* is the bin size and *e* is Euler’s number. Interestingly, we found that the increase in normalized entropy is much greater for a-thorny cells than for thorny ones (Fig 7G): the reason for this mainly resides in the different temporal structure of the spike trains of the two cells. Indeed, in the absence of clustered synaptic stimulation, a-thorny cells have a high coefficient of variation of the ISIs (around 1.3), but a low value of entropy (around 0.6 times the entropy of a Poisson process at the same firing rate, Fig 7G). This can be explained by the fact that in a bursting firing pattern each spike has a high probability of being followed by one or more additional spikes, and this reduces the “uncertainty” of the spike train, i.e., its entropy. Thorny cells, on the other hand, have lower values of CV (around 0.65), but the corresponding values of entropy are much higher and closer to the maximum achievable entropy (Fig 7G). A direct consequence of this is that when a-thorny cells are stimulated with an additional synaptic input, they can substantially increase the entropy of their output spike train and therefore have a greater capacity to encode additional information in their output spike train. This is confirmed by the greater increase in mutual information between the onset times of presynaptic bursts and the output spike times that can be observed in a-thorny cells (Fig 7H), further indicating that this cell type has a greater capacity for representing in its output spike train the arrival of correlated inputs. These results indicate that, because of their bursting firing pattern, a-thorny cells are more suited to transferring information about correlated inputs to their firing output, potentially operating as “detectors” for the presence of correlated activity in the CA3 network.

## Discussion

Similar to many other regions of the mammalian brain, the hippocampus is characterized by a striking variety of cell types [26,38,39]. For instance, we have previously shown that principal neurons in the CA3 area are subdivided in two classes: regular-spiking (RS) pyramidal cells with thorny excrescences and a previously uncharacterized a-thorny intrinsic-bursting (IB) cell type [21]. To further support the rationale for this subdivision, here we analyzed single-cell transcriptomic data from the CA3 region and found that clustering analysis—based on all genes as well as ion channel genes specifically—supports our hypothesis that there are two major principal cell types in the CA3 region (see Figs 1F, 3B and 3D). When interconnected in cell type-specific pathways, hippocampal circuits provide the basis for its rich repertoire of emergent dynamical patterns supporting cognitive function [40,41]. Among these are SWs, a network synchronization event implicated in both memory recall and consolidation [42]. SWs have been shown to originate in the CA3 region of the hippocampus [43,44], where a-thorny cells burst preferentially at the SW onset [21], thereby promoting synchronization. This raises an interesting question: which biophysical mechanisms and synaptic input patterns can drive this form of spiking output? Here, we adopted a data-driven approach employing a combination of patch-clamp electrophysiology and high-resolution morphological data, to build multi-compartmental, biophysically detailed models for each of the two CA3 principal cell populations. To this end, we employed multi-objective optimization methods [16], which have become the *de facto* standard for generating biophysically realistic models [17,18,20,45,46]. Compared with a hand-tuned model, a fully agnostic, data-driven approach to optimization might incur in the risk of choosing parameter values that are not biologically realistic, but that simply allow the model to achieve an adequate fitness level. This problem can be mitigated by choosing appropriate, possibly cell-type specific, intervals over which parameters are allowed to vary: in our case, we used a similar parameter set as the one described in [20] and chose appropriate intervals to make them suitable for CA3 pyramidal cells. Multi-objective optimization generates a set of viable solutions, termed individuals, that satisfy the experimental constraints to different degrees: by setting a threshold on the quality of the individuals, we were able to generate two cell populations that not only quantitatively match the firing phenotypes observed in individual CA3 RS and IB neurons, but that are also capable of reproducing the population-level variability in their intrinsic properties (Fig 2). Viewed in this perspective, the detailed biophysical models of CA3 principal neurons developed in this study constitute the first step towards the goal of gaining a mechanistic understanding of the biophysical mechanisms underlying the cellular contributions to network dynamics in the CA3 region.

### Distinct physiological and morphological features shape the cell type-specific phenotype

We compared the distributions of model parameters across the population of individuals generated by the optimization algorithm with the cell type-specific differences in expression levels of ion channel genes in our scRNAseq dataset and found several important congruencies, as shown in Fig 3. In particular, the most prominent differences between RS and IB cells in parameter distributions are related to K^+^ channels. Beyond the conventional delayed-rectifier conductance responsible for AP termination, both A-type and M-type K^+^ channels are down-regulated in the IB cell type, indicating a possible mechanism for somatic burst generation in these cells. These results are consistent with a previous modelling and experimental study [24], which highlighted the importance of M-type K^+^ potassium channel in burst generation in CA1 pyramidal cells. Additionally, we found that the parameters regulating intracellular Ca^2+^ dynamics are also distinct between the two cell types, in agreement with a previous experimental report [47]. These results constitute a first important step towards incorporating single-cell and population-level variability of excitatory neurons into detailed network models of the hippocampus, which so far have mostly focused on inhibitory neuron heterogeneity [48]. In addition to the parameter distribution differences generated by the multi-objective optimization procedure, thorny and a-thorny cells display several key anatomical differences [21] that contribute to each cell type intrinsic properties. To test the potential functional consequences of such differences on the processing capabilities of the two cell types, we investigated the effect of dendritic diameter on R_in_ and amplitude ratio differences between CA3 principal neurons, as shown in Fig 4. We found that, for a given dendritic diameter, a-thorny cells have on average higher R_in_ and AR than thorny cells; when looking specifically at terminal branches (Fig 4G and 4H), our modelling results indicate that the values of R_in_ and AR are approximately twice as large in a-thorny cells, albeit with decreasing separation as the distance from the parent dendritic branch increases. Given that the spine model was the same for the two cell types, this is largely attributable to the differences in axial resistance (which is greater in a-thorny cells) and hints at a possible higher compartmentalization of inputs arriving on the synapses of a-thorny cells. Our results are in agreement with several previous studies that have shown a high electrical compartmentalization of dendritic spines (see [49] for a review), even though recent imaging studies have hinted at a much lower degree of compartmentalization [50,51]. Further experimental work is therefore required to assess the true measure of spine head compartmentalization, given its potentially important functional consequences [49,52].

### Synaptic cooperativity

In vivo, neurons are constantly bombarded by synaptic inputs with varying spatio-temporal patterns. Understanding the basis of how synaptic inputs interact to drive nonlinearities and action potential output is of critical importance for elucidating the neuronal transfer function. To better understand how synaptic inputs interact in the two principal CA3 cell types as well as to maintain a data-driven approach, we used in vitro EPSP statistics recorded with and without Na^+^ channel blockers to constrain the AMPA and NMDA synapses added to the neuron models. In this experimental scenario, we investigated the role played by the number of activated synapses and the temporal patterns of activation [29] and found that, when Na^+^ channels’ conductance was set to 0, mimicking the bath application of the sodium channel blocker TTX, both cell types display a supralinear increase of the dendritic and somatic EPSPs (see Fig 5), which is a direct consequence of the strong input-output nonlinearity introduced by NMDARs. Furthermore, when setting the sodium conductances to the values obtained by the optimization procedure, we found that the concomitant activation of synaptic inputs on the apical dendrite of a-thorny, but not thorny cells, leads to a dendritic plateau resulting in somatic bursts, as shown in Fig 6. This occurs despite the fact that the target features used in the optimization procedure did not include any dendritic stimulus. Therefore, this behavior emerges “naturally” and points to the interplay between ionic and synaptic conductances in the dendrites and somatic conductances as a second source of bursting in the a-thorny cells, in addition to the more established slow-fast basis of bursting observed at the level of somatic currents [24]. To the best of our knowledge, this is the first time that a data-driven biophysically-detailed neuron model of a hippocampal cell type is capable of displaying dendritic plateau bursting (but see [53] for a pioneering hand-tuned model incorporating this feature).

### Mutual information and correlation transfer

One of the key features of CA3 that differentiates it from the rest of the hippocampus is its recurrent connectivity (see [54] for a review). In a final set of simulation experiments aimed at recapitulating spontaneous recurrent network activity observed in vitro and in vivo during SWs, we injected a fluctuating current into the soma of the simulated cells to mimic the concurrent activation of a large number of (uncorrelated) presynaptic neurons, while delivering the same synaptic activation pattern described previously, with varying degrees of spatio-temporal correlation. Strikingly, we found that the firing patterns of a-thorny cells have a lower entropy than their thorny counterparts: in turn, this allows this cell type to encode more information about their synaptic input in their spiking output, as quantified in Fig 7 by using the mutual information measure. This suggests that synapses on a-thorny cells have a higher information efficacy [36] thereby enabling this cell type to operate as “correlation detectors” that use high-frequency bursts of APs to signal the arrival of tightly clustered volleys of presynaptic inputs on their dendritic trees. It remains to be verified (both in silico and in vitro) whether there are any differences in “noise” correlation transfer between the two cell types, as has been shown for various types of neurons in rat somatosensory cortex [55]. Higher information-transfer capabilities might have profound functional implications for the role played by a-thorny cells in the hippocampal circuitry, as it could at least partially explain the preferential involvement of this cell type in the early phases of SWs [21]. It is worthwhile pointing out that the simulation experiments reported in Fig 7 have been performed at a fixed level of simulated background synaptic input: given that background synaptic activity is correlated with brain state (both at the micro- and macro-circuit levels), further work is required to elucidate the effect of varying degrees of noise on the amount of mutual information both cell types are capable of transferring by means of an interplay between high-conductance state [34] and stochastic resonance [56].

### Correlation transfer driven bursting behavior and CA3 network synchronization dynamics

Systems memory consolidation involves network oscillations known as sharp-wave ripples. These LFP fluctuations originate from the synchronization of ensembles of pyramidal cells in the CA3 region of the hippocampus and then propagate to CA1 and other extra-hippocampal brain regions. Previously we demonstrated that burst-firing—specifically from a-thorny CA3 pyramidal neurons—was significantly correlated with the onset of sharp-wave synchronization [21]. Moreover, we demonstrated in a spiking recurrent network model consisting of bursting and regular-spiking cell types, that bursts provide the essential nonlinearity around which the network can synchronize. Within this dynamical systems context, we postulated that information present in the correlational structure of the ongoing spontaneous activity in the network could be selectively amplified by a-thorny cells to promote network synchronization. In the present study we investigate how this type of nonlinear amplification is achieved and provide an information theoretic description for how correlation transfer is performed in a cell type specific manner. Within the context of CA3 recurrent network synchronization dynamics our results shed light on key biophysical mechanisms and synaptic input patterns that support complex-spiking output of a-thorny cells thereby providing deeper insight into the mechanisms of hippocampal sharp-wave initiation.

## Materials and methods

### Ethics statement

Experimental procedures adhered to methods approved by Cedars-Sinai Medical Center Institutional Animal Care and Use Committee.

### Acute brain slice electrophysiology and morphological reconstruction

Acute hippocampal brain slices were prepared from male and female mice (postnatal day (P) 23 to P40) according to [21]. Briefly, after animals were anesthetized with isoflurane, mice were decapitated and the brain rapidly removed and placed into chilled sucrose cutting solution consisting of (in mM) 215 sucrose, 2.5 KCl, 20 glucose, 26 NaHCO3, 1.6 NaH2PO4, 1 CaCl2, 4 MgCl2, and 4 MgSO4. Whole hippocampi were dissected out of the brain, embedded in a preformed agar block (4% agar), cut into 400-μm thick transverse sections on a Leica VT 1200 s vibratome, and transferred to a submersion incubation chamber containing room-temperature artificial cerebrospinal fluid (ACSF) containing (in mM) 124 NaCl, 2.5 KCl, 10 glucose, 26 NaHCO3, 1.0 NaH2PO4, 2.0 CaCl2, and 1.0 MgCl2. Both cutting and ACSF solutions were saturated with 95% O2 and 5% CO2 (pH 7.2–7.4) throughout slice preparation. The slices were incubated in ACSF for at least 1 h before recording, and then were transferred as needed to a submersion-type laminar-flow recording chamber, perfused with ACSF at 5 mL/min. Whole-cell intracellular recordings were obtained using standard patch-clamp techniques in current-clamp mode visualized under infrared differential interference contrast (IR-DIC) optics. The internal pipette solution for all recordings contained (in mM) 135 potassium-gluconate, 5 KCl, 1 CaCl2, 0.1 EGTA-Na, 10 HEPES, 10 glucose, 5 MgATP, 0.4 Na3GTP, and 0.5% biocytin, at pH 7.2 and 285–290 mOsm. To maximize cell health, synaptic connectivity, recording stability, and to obtain an unbiased sampling of CA3 pyramidal neurons, cells 100–150 μm below the surface of the slice were blind-patched. The recording pipette resistance ranged from 4–6 MΩ. Bridge balance and capacitance compensation were monitored and manually adjusted (as needed) throughout each recording. Recordings with > 20% changes in input resistance (R_in_) were systematically excluded from analysis. Resting potential ranged from –79 to –58 mV. Maximal recording time after dissection was 6 h. Recording temperature was set to 32.0 ± 0.1°C. Recordings were Bessel-filtered at 5 kHz and digitized at 20 kHz and analyzed using IgorPro or pClamp11 software. In all cases, a series of 500-ms current steps were applied to each cell, held at –60 ± 1 mV, within 5–10 min after break-in, to determine the intrinsic properties of the cell being recorded. Cells were classified as regular-spiking or intrinsically bursting by the initial spike frequency resulting from a suprathreshold current injection 10–20% above rheobase for regular-spiking cells and at rheobase for intrinsically bursting cells. A host of physiological parameters in addition to the initial firing frequency, R_in_, and rheobase, were analyzed consistently with previous measurements [21]. Neurons were filled with biocytin for at least 20 min, and slices were then fixed for a minimum of 12–24 h with 4% paraformaldehyde after recording. Fixed brain slices were then washed in 1 × PBS solution before staining. Biocytin staining was performed with vector PK4000 and SK4100 kits (Vector Laboratories, Burlingame, CA, USA). Digital images (z-stacks; 1-μm intervals) of biocytin-stained neurons were obtained using a Zeiss Axioimager upright microscope equipped with a 100 × (1.4 numerical aperture) objective. The z-stacks were then imported into the ShuTu software [57] for reconstruction and analysis.

### Transcriptomic analysis of single-cell RNA-sequencing data from CA3 excitatory neurons

We utilized scRNAseq data for 314 cells from the CA3 region of the hippocampus from the Allen institute for Brain Science (2018 Allen Institute for Brain Science. Cell Types Database: RNA-Seq Data. Available from portal.brain-map.org/atlases-and-data/rnaseq). A keyword search of “ion channel” for species *mus musculus* in the PANTHER database [58] yielded 417 relevant genes, 400 of which were mapped to the genome in our dataset. Focusing on this list of ion channel-related genes, single-cell gene expression analysis was performed using the SCANPY Python package (v1.7.0) [59] with any non-default parameters shown in Table 1. After normalizing the raw counts to counts per million (CPM) using the scanpy.pp.normalize_total function, the dimensionality of the data was reduced by running principal component analysis (PCA) with the scanpy.pp.pca function. Next, the neighborhood graph of cells was computed using the PCA representation of the data with the function scanpy.pp.neighbors. Uniform manifold approximation and projection (UMAP) [22] was used to embed the graph in two dimensions using the function scanpy.tl.umap. Finally, the neighborhood graph was clustered using the Leiden graph-clustering method through the function scanpy.tl.leiden (Fig 3C). Differential gene expression analysis was performed between these main clusters using the DESeq2 package in R [60] with default parameters. The most highly differentially expressed genes were extracted using a -log10(p-value) cut-off of 1e-7 and a log2(fold change) cut-off of 1.4 determined by inspection of the volcano plot (S2 Fig).

**Table 1:** SCANPY parameters used for analysis.

Function name	Parameter name	Parameter value
scanpy.pp.filter_genes	min_cells	3
scanpy.pp.normalize_total	target_sum	1e6
scanpy.pp.pca	exclude_highly_expressed	True
	n_comps	50
scanpy.pp.neighbors	n_neighbors	10
	n_pcs	50
scanpy.tl.umap	min_dist	0.05
scanpy.tl.leiden	resolution	0.1

### Optimization of biophysical models

In order to optimize the parameters of the models, we employed the BluePyOpt toolbox [17], which relies on the DEAP Python library [61]. To tune the parameter values, BluePyOpt implements the Indicator Based Evolutionary Algorithm (IBEA), which has been shown to perform particularly well on this kind of problem [62]. Briefly, the algorithm consists in solving a multi-objective optimization problem by evolving a population of solutions (termed “*individuals*”) that satisfy to different degrees a set of objectives. Similarly to previous optimization approaches [16,25], BluePyOpt employs as targets for the optimization features extracted from the V_m_ recorded in patch-clamp experiments rather than the time series of the V_m_ itself. All the models presented in this paper were obtained by using the following set of 12 features in the optimization process, extracted from the experimental data using the open source Electrophysiological Feature Extraction Library (eFEL, available at https://github.com/BlueBrain/eFEL): resting V_m_, AP amplitude, threshold, half-width and fall and rise rates, coefficient of variation (CV) of the inter-spike intervals (ISI), number of spikes emitted during a 500 ms-long stimulation, inverse of the first ISI, V_m_ at the after-hyperpolarization (AHP), times to first and last AP in the train. These were a slightly modified version of the ones employed in [20], but we found that the optimization results were robust to reasonable changes in the set of optimization features. Importantly, we used the same set of features for both cell types and let the optimization procedure choose the appropriate values for the parameters. The stimulation protocols consisted in three steps of depolarizing DC currents whose amplitudes were chosen to span the current range over which the experimental f-I curves were measured and were therefore different for the two cell types. For each individual in the population, BluePyOpt runs the model for the three values of DC current, extracts the feature values and computes the individual’s *fitness* value as the sum of the distance of each feature from the corresponding experimental mean in units of experimental standard deviation. At each optimization epoch, the best individuals (in terms of fitness) are selected and combined to create the next generation of solutions. The number of free parameters in the models was 24 and consisted of the maximal conductance values associated to each ion channel inserted in the models (18 total parameters) plus (i) the reversal potential for the leak current (E_L_), (ii) the axial resistance (Ra), (iii) the time constant of intracellular calcium dynamics and (iv) a parameter determining the availability of free calcium inside the cell [63]. The E_L_ and Ra parameters were optimized to have different values in the axon and in the rest of the cell. The set of active conductances used in our models is the same as that described in [20]. Additionally, in order to minimize the impact of the different degree of detail in axon reconstruction that was present in our morphologies, we substituted the original axon with a stereotypical one composed of two 30 μm-long compartments, modelling the axon initial segment [17]. Overall, we used a total of six morphologies (three per cell type) and, given the vastity of the 24-dimensional parameter space and the stochastic nature of evolutionary algorithms such as DEAP, we performed several optimization runs for each morphology.

### Simulations

Simulations were run in NEURON [64] using its Python interface [65] and the variable time step (CVODE) method. The fixed parameters were the reversal potentials of sodium, potassium, and calcium currents (set at 50, -90 and 130 mV, respectively), the membrane capacitance (set at 1 mF/cm^2^) and the simulation temperature (set at 34°C as in the experiments). The resting value of [Ca^2+^]_i_ was set at 50 nM. Optimizations were performed on a cluster of 32- or 48-core Intel Xeon processors running Scientific Linux 7.6 and typically took between 3 and 24 hours to complete, depending on the complexity of the morphology, i.e., on the total number of iso-potential compartments in which the model was discretized, and typically included 150 individuals that were evolved for 100 generations.

### Individual selection and parameter dimensionality reduction

One of the main advantages of using an evolutionary algorithm is that it provides a family of solutions, called *individuals*, that match to different degrees the optimization features (i.e., they lie on the Pareto frontier that makes up the optimal trade-off between satisfying different objectives [16]. Following each optimization run, we selected only those individuals that had an error for each feature below 6 units of standard deviation from the experimental mean: this led to a total of 180 individuals for the thorny cell type and 172 for the a-thorny one. To visualize the similarity across distinct individuals, we performed dimensionality reduction on the 24 parameters using UMAP [22], using the code available at https://github.com/lmcinnes/umap. Parameter values were converted to their z-score using the StandardScaler of scikit-learn [66] before applying each dimensionality reduction algorithm.

### Spine model

Spines were modelled in NEURON as two cylindrical compartments connected to the dendritic tree: the first one, representing the spine neck, had length and diameter of 1.58 and 0.077 μm, respectively, while the second one had both length and diameter equal to 0.5 μm (corresponding to an external surface equivalent to that of a sphere of 0.5 μm diameter) [29]. The axial resistivity of the spine was the same as that of the compartment to which it was connected and therefore assumed different values depending on the individual that was simulated (ranging from values around 150 Ω∙cm for the thorny cell type to approximately 340 Ω∙cm for the a-thorny one). Spines were added only in the experiments related to the computation of dendritic amplitude ratio and synaptic cooperativity. No modifications to the membrane capacitance were applied due to the limited number of spines (up to 9, spaced 5 μm apart) placed simultaneously on the dendritic tree. In the experiments where spines were present, the distribution of sodium channels in the apical dendrites of the a-thorny cell type was modified to have an exponential decay from 100% to 50% of the somatic value (in order not to have discontinuities), with a length constant of 100 μm.

### Dendritic input resistance computation

Dendritic R_in_ was computed by injecting 500 ms-long hyperpolarizing pulses of current of amplitude -50 pA and measuring the V_m_ deflection at the end of the pulse. For the experiments shown in Fig 4, this was done for every compartment of the model and then plotted with respect to either the compartment distance on the dendritic branch or the dendrite diameter.

### Amplitude ratio computation

To compute the amplitude ratio shown in Fig 4, we injected EPSP-shaped currents (modelled as a double exponential function with rise and decay times equal to 1 and 10 ms, respectively) in the spine head and measured the elicited EPSP both in the spine and in the dendrite directly connected to it (which we term EPSP_spine_ and EPSP_dend_, respectively). The amplitude ratio AR is therefore given by

$$AR=\frac{EPSP_{spine}}{EPSP_{dend}}$$ (1)

and constitutes a measure of the degree of compartmentalization that spines provide on the incoming inputs. Following [29], given AR and R_dend_, the dendrite input resistance, we estimated the input resistance of the spine neck as

$$R_{neck}=(AR-1)R_{dend}$$ (2)

In the synaptic cooperativity experiments shown in Fig 5, the AR was computed as in Eq 2, with the only difference that the spine EPSP was elicited by the activation of a presynaptic event rather than by the injection of an EPSP-shaped current. The range of AR values we observed when activating spines is comparable to what is obtained with the injection of EPSP-shaped currents.

### Synapse models

In the synaptic cooperativity and mutual information experiments, spines contained AMPA and NMDA receptors. Both AMPA and NMDA synapses were modelled as bi-exponential functions: the former had rise and decay time constants of 0.1 and 1 ms, respectively [29], while the latter had rise and decay time constants equal to 1 and 50 ms for the thorny cell and 1 and 100 ms for the a-thorny one. This was done to account for the longer decay of EPSPs observed in the a-thorny cell type (S3 Fig). For NMDA synapses, the removal of the magnesium block was modelled according to [67,68] by multiplying the NMDA conductance by the following coefficient:

$$\frac{1}{1+\frac{{\left[Mg\right]}_{o}}{K_{d}}\exp(\gamma (sh-V_{m}))}$$ (3)

where [Mg]_o_ is the external magnesium concentration (set at 1 mM) and K_d_ = 9.888 mM, sh = -7.778 mV and γ = 2.222 V^-1^ are parameters that regulate the V_m_ dependence of the removal of magnesium from the synaptic cleft. The synaptic weights were chosen to have a V_m_ deflection at the spine head of approximately 20 mV when only one presynaptic event was simulated, in line with previous modelling studies [29].

### Computation of currentscapes

Currentscapes were computed using the algorithm presented in [32]: briefly, positive (outward) or negative (inward) ionic currents were organized as rows in a matrix, with each column representing a time instant. Total outward or inward currents (filled black areas in the middle and bottom panels of Fig 6A and 6B) were then computed as the row-wise sum of the elements in the matrixes, while the percent contribution of each ionic current was obtained by further dividing each row in the matrix by the corresponding total current and multiplying this value by 100.

### Computation of mutual information

Several works have investigated how to compute the mutual information between spike trains [36,69–72] and V_m_ traces [73,74]. Here, we used the same approach employed by [36], which is based on the context-tree weighting (CTW) method first described in [37,75]. Briefly, having first converted a spike train into a binary string ***x***∈{0,1}^*n*^ of length *n* using an appropriate bin size (4 ms in our case), the context-tree weighting method provides an estimate $\hat{P}$ of the *probability* of the string ***x*** over all possible independent and identically distributed (i.i.d.) stochastic sources that can generate it. The estimate of the entropy of the string ***x*** is therefore given by

$${\hat{H}}_{n}(x)≜-\frac{1}{n}\log\hat{P}(x)$$ (4)

which is measured in bits/symbol or bits/s if the symbol rate is known. The advantage of using the CTW method is that it provides reliable estimates of the probability of a binary string even when its length is relatively short (a few hundreds of APs are generally sufficient), in contrast to the direct method, which requires unreasonably long spike trains to provide a good estimate. The CTW method can also be used to compute the mutual information *MI* between two binary sequences ***x*** and ***y***. To do so, one must rely on the well-known definition of the mutual information:

MI(x;y)=H(x)−H(x|y) (5)

where *H*(***x***|***y***) is the entropy of the sequence ***x*** given that the sequence ***y*** is known. A detailed description on how to obtain the MI estimate can be found in the supplementary materials of [36].

## Supporting information

S1 Fig Violin plots of the distributions of the parameter values not shown in Fig 3 for the model cells included in the analysis, normalized over the range of allowed variability of each parameter (black and red indicate thorny and a-thorny cells, respectively). Dashed lines indicate the median of the population, while the upper and lower dotted lines represent the 25th and 75th percentile of the distributions (significant differences tested with a non-parametric Kolmogorov-Smirnov test: * p < 0.05, ** p < 0.01, *** p < 0.001).
(TIF)

S2 Fig Volcano plot for gene expression levels of cells in cluster 1 (putative thorny cells) vs. cells in cluster 2 (putative a-thorny cells). Each marker represents a gene: purple markers indicate genes with a significantly different gene expression that is above the fold change threshold level. Among these, genes marked with cyan dots are ion channel genes.
(TIF)

S3 Fig Left: violin plots of the distributions of amplitude, rise time constant and decay time constant of spontaneous EPSPs recorded in thorny and a-thorny cells (black and red violins, respectively) during bath application of the Na channel blocker TTX (one-way Welch ANOVA with one-step Bonferroni correction for multiple comparisons: * p < 0.05, *** p < 0.001). Right: voltage traces used for the extraction of EPSP parameters (pink and gray traces are individual EPSPs, while black and red traces are averages for thorny and a-thorny cells, respectively).
(TIF)

S1 Table Electrophysiological properties of thorny and a-thorny cells measured in vitro. (DOCX)

S2 Table Parameter names as shown in Figs 3 and S1 with corresponding channel description, location on the cell morphology and allowed bounds of variation during optimization. (DOCX)

## References

[1] Lisman J. Bursts as a unit of neural information: making unreliable synapses reliable. Trends Neurosci. 1997;20: 38–43. doi: 10.1016/S0166-2236(96)10070-9 9004418

[2] Izhikevich EM, Desai NS, Walcott EC, Hoppensteadt FC. Bursts as a unit of neural information: selective communication via resonance. Trends Neurosci. 2003;26: 161–167. doi: 10.1016/S0166-2236(03)00034-1 12591219

[3] Zeldenrust F, Wadman WJ, Englitz B. Neural Coding With Bursts—Current State and Future Perspectives. Front Comput Neurosci. 2018;12. doi: 10.3389/fncom.2018.00048 30034330 PMC6043860

[4] Harnett MT, Bernier BE, Ahn K-C, Morikawa H. Burst-Timing-Dependent Plasticity of NMDA Receptor-Mediated Transmission in Midbrain Dopamine Neurons. Neuron. 2009;62: 826–838. doi: 10.1016/j.neuron.2009.05.011 19555651 PMC2702773

[5] Hunt DL, Puente N, Grandes P, Castillo PE. Bidirectional NMDA receptor plasticity controls CA3 output and heterosynaptic metaplasticity. Nat Neurosci. 2013;16: 1049–1059. doi: 10.1038/nn.3461 23852115 PMC3740388

[6] Takahashi H, Magee JC. Pathway Interactions and Synaptic Plasticity in the Dendritic Tuft Regions of CA1 Pyramidal Neurons. Neuron. 2009;62: 102–111. doi: 10.1016/j.neuron.2009.03.007 19376070

[7] Bittner KC, Milstein AD, Grienberger C, Romani S, Magee JC. Behavioral time scale synaptic plasticity underlies CA1 place fields. Science. 2017;357: 1033–1036. doi: 10.1126/science.aan3846 28883072 PMC7289271

[8] Bittner KC, Grienberger C, Vaidya SP, Milstein AD, Macklin JJ, Suh J, et al. Conjunctive input processing drives feature selectivity in hippocampal CA1 neurons. Nat Neurosci. 2015;18: 1133–1142. doi: 10.1038/nn.4062 26167906 PMC4888374

[9] Zhao X, Hsu C-L, Spruston N. Rapid synaptic plasticity contributes to a learned conjunctive code of position and choice-related information in the hippocampus. Neuron. 2022;110: 96–108. doi: 10.1016/j.neuron.2021.10.003 34678146

[10] Zhao X, Wang Y, Spruston N, Magee JC. Membrane potential dynamics underlying context-dependent sensory responses in the hippocampus. Nat Neurosci. 2020;23: 881–891. doi: 10.1038/s41593-020-0646-2 32451487

[11] De Schutter E, Bower JM. An active membrane model of the cerebellar Purkinje cell. I. Simulation of current clamps in slice. J Neurophysiol. 1994;71: 375–400. doi: 10.1152/jn.1994.71.1.375 7512629

[12] Lazarewicz MT, Migliore M, Ascoli GA. A new bursting model of CA3 pyramidal cell physiology suggests multiple locations for spike initiation. Biosystems. 2002;67: 129–137. doi: 10.1016/s0303-2647(02)00071-0 12459292

[13] Mainen ZF, Sejnowski TJ. Influence of dendritic structure on firing pattern in model neocortical neurons. Nature. 1996;382: 363–366. doi: 10.1038/382363a0 8684467

[14] Migliore M, Cook EP, Jaffe DB, Turner DA, Johnston D. Computer simulations of morphologically reconstructed CA3 hippocampal neurons. J Neurophysiol. 1995;73: 1157–68. doi: 10.1152/jn.1995.73.3.1157 7608762

[15] Traub RD, Wong RK, Miles R, Michelson H. A model of a CA3 hippocampal pyramidal neuron incorporating voltage-clamp data on intrinsic conductances. J Neurophysiol. 1991;66: 635–650. doi: 10.1152/jn.1991.66.2.635 1663538

[16] Druckmann S, Banitt Y, Gidon A, Schurmann F, Markram H, Segev I. A novel multiple objective optimization framework for constraining conductance-based neuron models by experimental data. Front Neurosci. 2007;1: 7–18. doi: 10.3389/neuro.01.1.1.001.2007 18982116 PMC2570085

[17] Van Geit W, Gevaert M, Chindemi G, Rossert C, Courcol JD, Muller EB, et al. BluePyOpt: Leveraging Open Source Software and Cloud Infrastructure to Optimise Model Parameters in Neuroscience. Front Neuroinform. 2016;10. doi: 10.3389/fninf.2016.00017 27375471 PMC4896051

[18] Gouwens NW, Berg J, Feng D, Sorensen SA, Zeng H, Hawrylycz MJ, et al. Systematic generation of biophysically detailed models for diverse cortical neuron types. Nat Commun. 2018;9: 710. doi: 10.1038/s41467-017-02718-3 29459718 PMC5818534

[19] Hay E, Hill S, Schürmann F, Markram H, Segev I. Models of Neocortical Layer 5b Pyramidal Cells Capturing a Wide Range of Dendritic and Perisomatic Active Properties. PLoS Comput Biol. 2011;7: e1002107. doi: 10.1371/journal.pcbi.1002107 21829333 PMC3145650

[20] Migliore R, Lupascu CA, Bologna LL, Romani A, Courcol JD, Antonel S, et al. The physiological variability of channel density in hippocampal CA1 pyramidal cells and interneurons explored using a unified data-driven modeling workflow. PLoS Comput Biol. 2018;14: e1006423. doi: 10.1371/journal.pcbi.1006423 30222740 PMC6160220

[21] Hunt DL, Linaro D, Si B, Romani S, Spruston N. A novel pyramidal cell type promotes sharp-wave synchronization in the hippocampus. Nat Neurosci. 2018;21: 985–995. doi: 10.1038/s41593-018-0172-7 29915194

[22] Becht E, McInnes L, Healy J, Dutertre CA, Kwok IWH, Ng LG, et al. Dimensionality reduction for visualizing single-cell data using UMAP. Nat Biotechnol. 2019;37: 38–44. doi: 10.1038/nbt.4314 30531897

[23] Hemond P, Epstein D, Boley A, Migliore M, Ascoli GA, Jaffe DB. Distinct classes of pyramidal cells exhibit mutually exclusive firing patterns in hippocampal area CA3b. Hippocampus. 2008;18: 411–24. doi: 10.1002/hipo.20404 18189311 PMC4339291

[24] Golomb D, Yue C, Yaari Y. Contribution of persistent Na+ current and M-type K+ current to somatic bursting in CA1 pyramidal cells: combined experimental and modeling study. J Neurophysiol. 2006;96: 1912–26. doi: 10.1152/jn.00205.2006 16807352

[25] Druckmann S, Berger TK, Hill S, Schurmann F, Markram H, Segev I. Evaluating automated parameter constraining procedures of neuron models by experimental and surrogate data. Biol Cybern. 2008;99: 371–9. doi: 10.1007/s00422-008-0269-2 19011925

[26] Yao Z, van Velthoven CTJ, Nguyen TN, Goldy J, Sedeno-Cortes AE, Baftizadeh F, et al. A taxonomy of transcriptomic cell types across the isocortex and hippocampal formation. Cell. 2021;184: 3222–3241.e26. doi: 10.1016/j.cell.2021.04.021 34004146 PMC8195859

[27] Traag VA, Waltman L, van Eck NJ. From Louvain to Leiden: guaranteeing well-connected communities. Sci Rep. 2019;9: 5233. doi: 10.1038/s41598-019-41695-z 30914743 PMC6435756

[28] Sherwood WE, Guckenheimer J. Dissecting the Phase Response of a Model Bursting Neuron. SIAM J Appl Dyn Syst. 2010;9: 659–703. doi: 10.1137/090773519

[29] Harnett MT, Makara JK, Spruston N, Kath WL, Magee JC. Synaptic amplification by dendritic spines enhances input cooperativity. Nature. 2012;491: 599–602. doi: 10.1038/nature11554 23103868 PMC3504647

[30] Makara JK, Magee JC. Variable Dendritic Integration in Hippocampal CA3 Pyramidal Neurons. Neuron. 2013;80: 1438–1450. doi: 10.1016/j.neuron.2013.10.033 24360546 PMC3878388

[31] Raus Balind S, Magó Á, Ahmadi M, Kis N, Varga-Németh Z, Lőrincz A, et al. Diverse synaptic and dendritic mechanisms of complex spike burst generation in hippocampal CA3 pyramidal cells. Nat Commun. 2019;10: 1859. doi: 10.1038/s41467-019-09767-w 31015414 PMC6478939

[32] Alonso LM, Marder E. Visualization of currents in neural models with similar behavior and different conductance densities. eLife. 2019;8: e42722. doi: 10.7554/eLife.42722 30702427 PMC6395073

[33] Uhlenbeck GE, Ornstein LS. On the theory of the Brownian motion. Phys Rev. 1930;36: 823.

[34] Destexhe A, Rudolph M, Paré D. The high-conductance state of neocortical neurons in vivo. Nat Rev Neurosci. 2003;4: 739–751. doi: 10.1038/nrn1198 12951566

[35] Fellous J-M, Rudolph M, Destexhe A, Sejnowski TJ. Synaptic background noise controls the input/output characteristics of single cells in an in vitro model of in vivo activity. Neuroscience. 2003;122: 811–829. doi: 10.1016/j.neuroscience.2003.08.027 14622924 PMC2928821

[36] London M, Schreibman A, Hausser M, Larkum ME, Segev I. The information efficacy of a synapse. Nat Neurosci. 2002;5: 332–40. doi: 10.1038/nn826 11896396

[37] Willems FMJ, Shtarkov YM, Tjalkens TJ. The Context-Tree Weighting Method—Basic Properties. IEEE Trans Inf Theory. 1995;41: 653–664. doi: 10.1109/18.382012

[38] Cembrowski MS, Spruston N. Heterogeneity within classical cell types is the rule: lessons from hippocampal pyramidal neurons. Nat Rev Neurosci. 2019;20: 193–204. doi: 10.1038/s41583-019-0125-5 30778192

[39] Klausberger T, Somogyi P. Neuronal Diversity and Temporal Dynamics: The Unity of Hippocampal Circuit Operations. Science. 2008;321: 53–57. doi: 10.1126/science.1149381 18599766 PMC4487503

[40] Buzsáki G. Rhythms of the Brain. Oxford University Press; 2006. doi: 10.1093/acprof:oso/9780195301069.001.0001

[41] Eichenbaum H. Hippocampus. Neuron. 2004;44: 109–120. doi: 10.1016/j.neuron.2004.08.028 15450164

[42] Buzsáki G. Hippocampal sharp wave-ripple: A cognitive biomarker for episodic memory and planning. Hippocampus. 2015;25: 1073–1188. doi: 10.1002/hipo.22488 26135716 PMC4648295

[43] Csicsvari J, Hirase H, Mamiya A, Buzsáki G. Ensemble Patterns of Hippocampal CA3-CA1 Neurons during Sharp Wave–Associated Population Events. Neuron. 2000;28: 585–594. doi: 10.1016/s0896-6273(00)00135-5 11144366

[44] Wittner L, Miles R. Factors defining a pacemaker region for synchrony in the hippocampus: CA3a *versus* CA3b in hippocampal synchrony. J Physiol. 2007;584: 867–883. doi: 10.1113/jphysiol.2007.138131 17823211 PMC2276992

[45] Iavarone E, Yi J, Shi Y, Zandt B-J, O’Reilly C, Van Geit W, et al. Experimentally-constrained biophysical models of tonic and burst firing modes in thalamocortical neurons. Lytton WW, editor. PLOS Comput Biol. 2019;15: e1006753. doi: 10.1371/journal.pcbi.1006753 31095552 PMC6541309

[46] Nandi A, Chartrand T, Geit WV, Buchin A, Yao Z, Lee SY, et al. Single-neuron models linking electrophysiology, morphology and transcriptomics across cortical cell types. Neuroscience; 2020 Apr. doi: 10.1101/2020.04.09.030239

[47] Roussel C, Erneux T, Schiffmann SN, Gall D. Modulation of neuronal excitability by intracellular calcium buffering: from spiking to bursting. Cell Calcium. 2006;39: 455–66. doi: 10.1016/j.ceca.2006.01.004 16530827

[48] Bezaire MJ, Raikov I, Burk K, Vyas D, Soltesz I. Interneuronal mechanisms of hippocampal theta oscillations in a full-scale model of the rodent CA1 circuit. eLife. 2016;5: e18566. doi: 10.7554/eLife.18566 28009257 PMC5313080

[49] Yuste R. Electrical Compartmentalization in Dendritic Spines. Annu Rev Neurosci. 2013;36: 429–449. doi: 10.1146/annurev-neuro-062111-150455 23724997

[50] Popovic MA, Carnevale N, Rozsa B, Zecevic D. Electrical behaviour of dendritic spines as revealed by voltage imaging. Nat Commun. 2015;6: 8436. doi: 10.1038/ncomms9436 26436431 PMC4594633

[51] Tønnesen J, Katona G, Rózsa B, Nägerl UV. Spine neck plasticity regulates compartmentalization of synapses. Nat Neurosci. 2014;17: 678–685. doi: 10.1038/nn.3682 24657968

[52] Araya R, Vogels TP, Yuste R. Activity-dependent dendritic spine neck changes are correlated with synaptic strength. Proc Natl Acad Sci. 2014;111: E2895–E2904. doi: 10.1073/pnas.1321869111 24982196 PMC4104910

[53] Traub RD, Jefferys JG, Miles R, Whittington MA, Tóth K. A branching dendritic model of a rodent CA3 pyramidal neurone. J Physiol. 1994;481: 79–95. doi: 10.1113/jphysiol.1994.sp020420 7853251 PMC1155867

[54] Le Duigou C, Simonnet J, Teleñczuk MT, Fricker D, Miles R. Recurrent synapses and circuits in the CA3 region of the hippocampus: an associative network. Front Cell Neurosci. 2014;7. doi: 10.3389/fncel.2013.00262 24409118 PMC3884140

[55] Linaro D, Ocker GK, Doiron B, Giugliano M. Correlation Transfer by Layer 5 Cortical Neurons Under Recreated Synaptic Inputs *In Vitro*. J Neurosci. 2019;39: 7648–7663. doi: 10.1523/JNEUROSCI.3169-18.2019 31346031 PMC6764207

[56] Mitaim S, Kosko B. Adaptive Stochastic Resonance in Noisy Neurons Based on Mutual Information. IEEE Trans Neural Netw. 2004;15: 1526–1540. doi: 10.1109/TNN.2004.826218 15565779

[57] Jin DZ, Zhao T, Hunt DL, Tillage RP, Hsu C-L, Spruston N. ShuTu: Open-Source Software for Efficient and Accurate Reconstruction of Dendritic Morphology. Front Neuroinformatics. 2019;13: 68. doi: 10.3389/fninf.2019.00068 31736735 PMC6834530

[58] Mi H, Ebert D, Muruganujan A, Mills C, Albou L-P, Mushayamaha T, et al. PANTHER version 16: a revised family classification, tree-based classification tool, enhancer regions and extensive API. Nucleic Acids Res. 2021;49: D394–D403. doi: 10.1093/nar/gkaa1106 33290554 PMC7778891

[59] Wolf FA, Angerer P, Theis FJ. SCANPY: large-scale single-cell gene expression data analysis. Genome Biol. 2018;19: 15. doi: 10.1186/s13059-017-1382-0 29409532 PMC5802054

[60] Love MI, Huber W, Anders S. Moderated estimation of fold change and dispersion for RNA-seq data with DESeq2. Genome Biol. 2014;15: 550. doi: 10.1186/s13059-014-0550-8 25516281 PMC4302049

[61] Fortin FA, De Rainville FM, Gardner MA, Parizeau M, Gagne C. DEAP: Evolutionary Algorithms Made Easy. J Mach Learn Res. 2012;13: 2171–2175.

[62] Markram H, Muller E, Ramaswamy S, Reimann MW, Abdellah M, Sanchez CA, et al. Reconstruction and Simulation of Neocortical Microcircuitry. Cell. 2015;163: 456–492. doi: 10.1016/j.cell.2015.09.029 26451489

[63] Destexhe A, Mainen ZF, Sejnowski TJ. Synthesis of models for excitable membranes, synaptic transmission and neuromodulation using a common kinetic formalism. J Comput Neurosci. 1994;1: 195–230. doi: 10.1007/BF00961734 8792231

[64] Carnevale NT, Hines ML. The NEURON book. Cambridge University Press; 2006.

[65] Hines ML, Davison AP, Muller E. NEURON and Python. Front Neuroinform. 2009;3: 1. doi: 10.3389/neuro.11.001.2009 19198661 PMC2636686

[66] Pedregosa F, Varoquaux G, Gramfort A, Michel V, Thirion B, Grisel O, et al. Scikit-learn: Machine Learning in Python. J Mach Learn Res. 2011;12: 2825–2830.

[67] Jahr CE, Stevens CF. Voltage dependence of NMDA-activated macroscopic conductances predicted by single-channel kinetics. J Neurosci. 1990;10: 3178–82. doi: 10.1523/JNEUROSCI.10-09-03178.1990 1697902 PMC6570236

[68] Jahr CE, Stevens CF. A quantitative description of NMDA receptor-channel kinetic behavior. J Neurosci. 1990;10: 1830–7. doi: 10.1523/JNEUROSCI.10-06-01830.1990 1693952 PMC6570302

[69] Dorval AD. Probability distributions of the logarithm of inter-spike intervals yield accurate entropy estimates from small datasets. J Neurosci Methods. 2008;173: 129–139. doi: 10.1016/j.jneumeth.2008.05.013 18620755 PMC2610469

[70] Houghton C. Calculating the Mutual Information between Two Spike Trains. Neural Comput. 2019;31: 330–343. doi: 10.1162/neco_a_01155 30576614

[71] Houghton C. Calculating mutual information for spike trains and other data with distances but no coordinates. R Soc Open Sci. 2015;2. ARTN 140391 doi: 10.1098/rsos.140391 26064650 PMC4453244

[72] Paninski L. Estimation of entropy and mutual information. Neural Comput. 2003;15: 1191–1253. doi: 10.1162/089976603321780272

[73] Fuhrmann G, Segev I, Markram H, Tsodyks M. Coding of temporal information by activity-dependent synapses. J Neurophysiol. 2002;87: 140–148. doi: 10.1152/jn.00258.2001 11784736

[74] Testa-Silva G, Verhoog MB, Linaro D, de Kock CP, Baayen JC, Meredith RM, et al. High bandwidth synaptic communication and frequency tracking in human neocortex. PLoS Biol. 2014;12: e1002007. doi: 10.1371/journal.pbio.1002007 25422947 PMC4244038

[75] Willems FMJ. The context-tree weighting method: Extensions. IEEE Trans Inf Theory. 1998;44: 792–798. doi: 10.1109/18.661523

