# Supplementary figures and images for: Cell type-specific mechanisms of information transfer in data-driven biophysical models of hippocampal CA3 principal neurons
Source: PLoS Comput Biol. 2022 Apr 22;18(4):e1010071. doi: 10.1371/journal.pcbi.1010071 (PMC9089861; doi:10.1371/journal.pcbi.1010071)

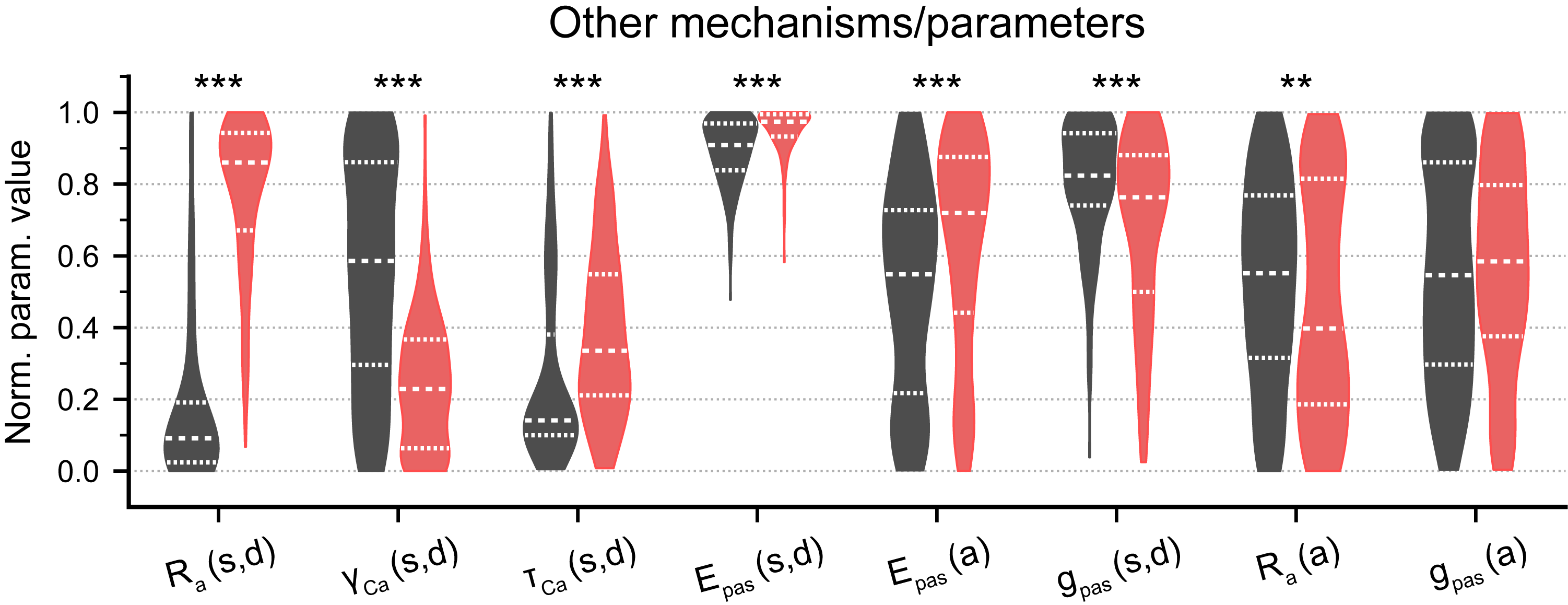

Supplement: S1 Fig — Dashed lines indicate the median of the population, while the upper and lower dotted lines represent the 25th and 75th percentile of the distributions (significant differences tested with a non-parametric Kolmogorov-Smirnov test: * p < 0.05, ** p < 0.01, *** p < 0.001). (TIF) [file pcbi.1010071.s001.tif]

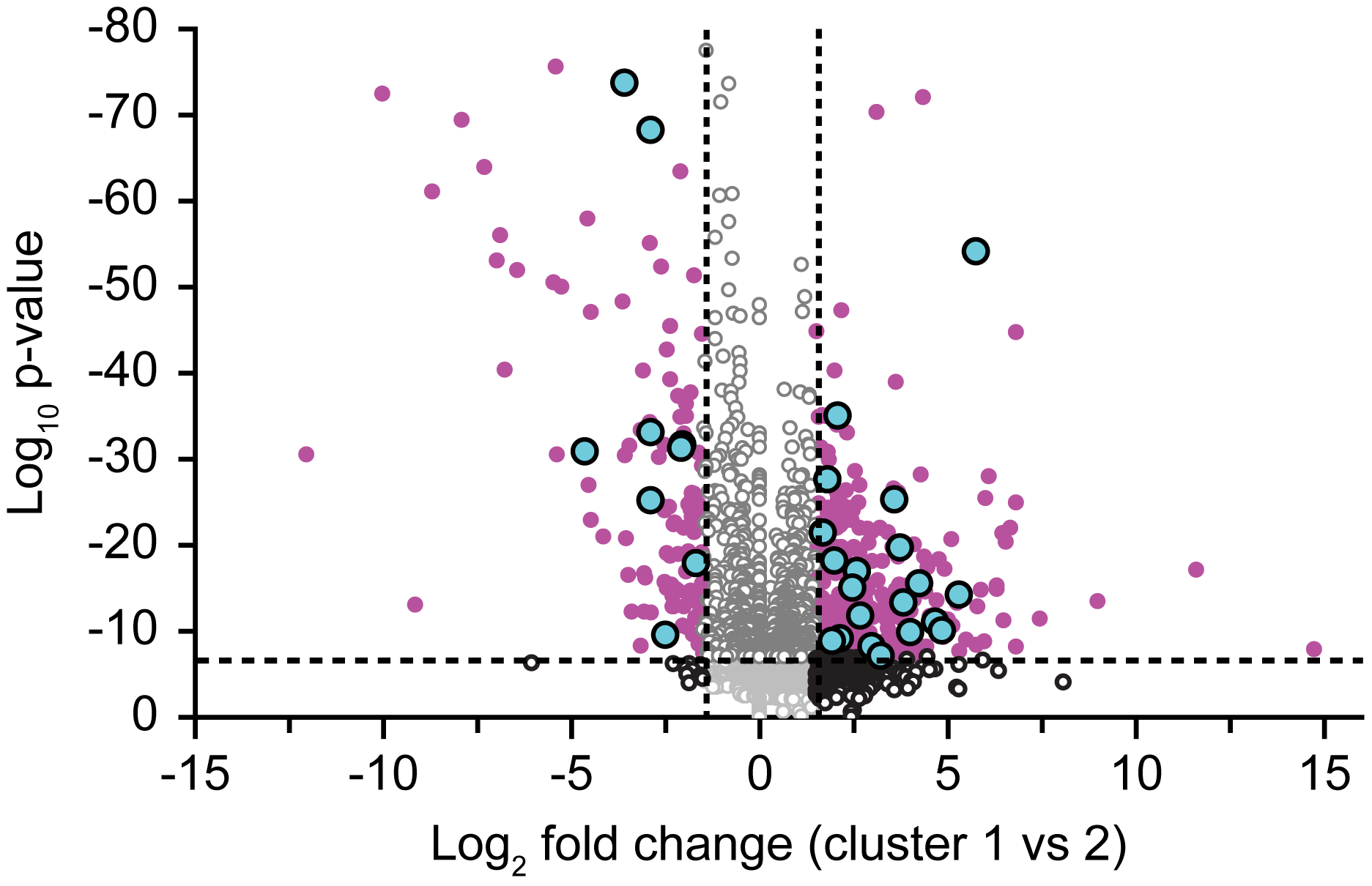

Supplement: S2 Fig — Each marker represents a gene: purple markers indicate genes with a significantly different gene expression that is above the fold change threshold level. Among these, genes marked with cyan dots are ion channel genes. (TIF) [file pcbi.1010071.s002.tif]

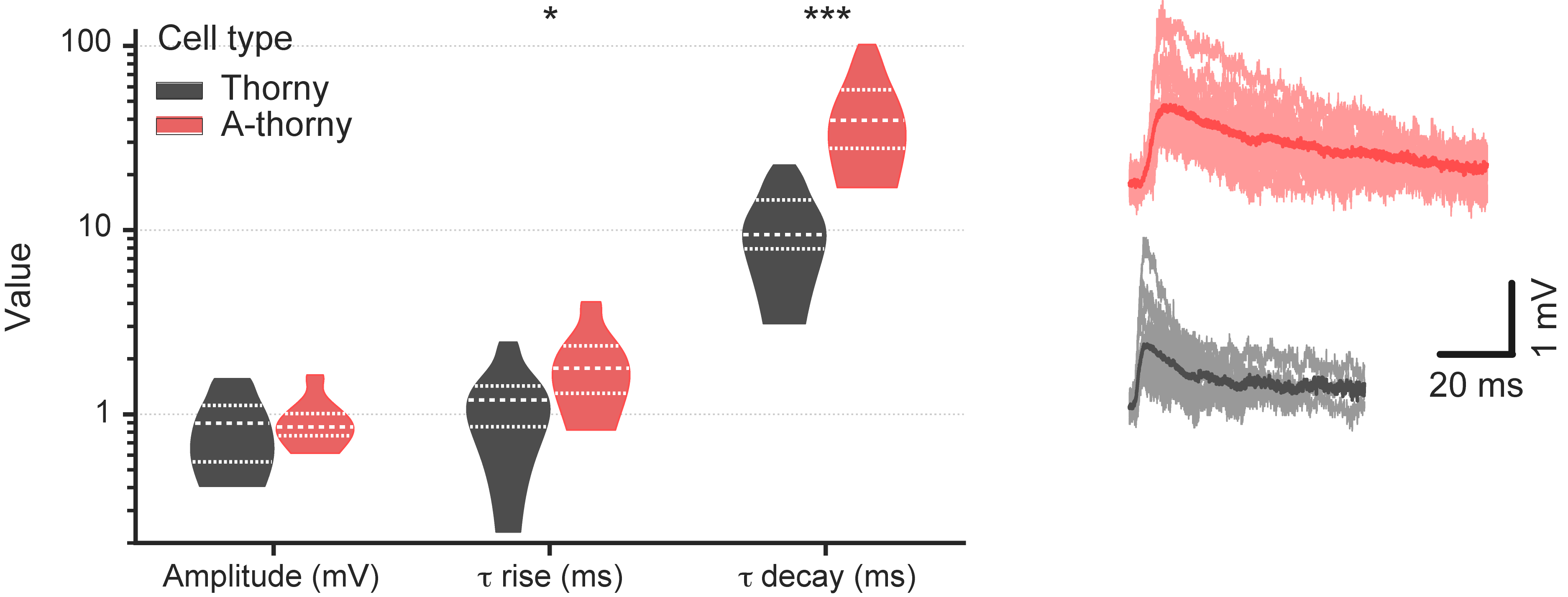

Supplement: S3 Fig — Right: voltage traces used for the extraction of EPSP parameters (pink and gray traces are individual EPSPs, while black and red traces are averages for thorny and a-thorny cells, respectively). (TIF) [file pcbi.1010071.s003.tif]
