# Supplementary material for: Cell type-specific mechanisms of information transfer in data-driven biophysical models of hippocampal CA3 principal neurons
Source: PLoS Comput Biol. 2022 Apr 22;18(4):e1010071. doi: 10.1371/journal.pcbi.1010071 (PMC9089861; doi:10.1371/journal.pcbi.1010071)
Supplement: S1 Table — (DOCX) [file pcbi.1010071.s004.docx]

|  | **Thorny** | | | | **A-thorny** | | | |
| --- | --- | --- | --- | --- | --- | --- | --- | --- |
|  | **Median** | **25th** | **75th** | **Range** | **Median** | **25th** | **75th** | **Range** |
| Initial frequency (spike/s) | 5.7 | 4.2 | 7.8 | 2.2,59.2 | 143 | 123 | 172 | 91,200 |
| Input resistance (MΩ) | 78 | 50 | 109 | 15,195 | 129 | 109 | 150 | 35,207 |
| ADP (mV) | -1 | -2.3 | 0.3 | -5.2,3.9 | 2.4 | 1.8 | 3.4 | -0.8,7.8 |
| AHP (mV) | -6.2 | -8.9 | -4.1 | -14.6,0.5 | -1.2 | -2.9 | -0.5 | -7.6,1.7 |
| Rheobase (pA) | 250 | 150 | 400 | 50,700 | 100 | 50 | 150 | 20,500 |
| First spike threshold (mV) | -44 | -48 | -39 | -56,-23 | -50 | -53 | -47 | -64,-32 |

Supplementary Table 1. Electrophysiological properties of thorny and a-thorny cells measured in vitro.
