# Supplementary material for: Cell type-specific mechanisms of information transfer in data-driven biophysical models of hippocampal CA3 principal neurons
Source: PLoS Comput Biol. 2022 Apr 22;18(4):e1010071. doi: 10.1371/journal.pcbi.1010071 (PMC9089861; doi:10.1371/journal.pcbi.1010071)
Supplement: S2 Table — (DOCX) [file pcbi.1010071.s005.docx]

| **Name** | **Description** | **Location** | **Bounds** | **Units** |
| --- | --- | --- | --- | --- |
| K_dr_ | Max. cond. of delayed-rectifier K channel | Axon | 0.01, 0.04 | S∙cm^-2^ |
|  |  | Soma, dendrites | 0.001, 0.008 | S∙cm^-2^ |
| K_A,p_ | Max. cond. of proximal A-type K channel | Axon | 0.025, 0.3 | S∙cm^-2^ |
|  |  | Soma | 0.025, 0.1 | S∙cm^-2^ |
| K_A,d_ | Max. cond. of distal A-type K channel | Dendrites | 0.005, 0.05 | S∙cm^-2^ |
| K_M_ | Max. cond. of M-type K channel | Axon | 0.002, 0.06 | S∙cm^-2^ |
|  |  | Soma | 0.0015, 0.06 | S∙cm^-2^ |
| K_Ca,s_ | Max. cond. of slow Ca-dependent K channel | Soma, dendrites | 2x10^-5^, 2x10^-4^ | S∙cm^-2^ |
| K_Ca_ | Max. cond. of Ca-activated K channel | Soma, dendrites | 10^-6^, 4x10^-4^ | S∙cm^-2^ |
| Ca_T_ | Max. cond. of T-type Ca channel | Soma, dendrites | 10^-6^, 2x10^-5^ | S∙cm^-2^ |
| Ca_L_ | Max. cond. of L-type Ca channel | Soma, dendrites | 10^-6^, 2x10^-5^ | S∙cm^-2^ |
| Ca_N_ | Max. cond. of N-type Ca channel | Soma, dendrites | 10^-6^, 2x10^-5^ | S∙cm^-2^ |
| Na_f_ | Max. cond. of fast inactivating Na channel | Axon | 0.1, 0.4 | S∙cm^-2^ |
|  |  | Soma, dendrites | 0.02, 0.08 | S∙cm^-2^ |
| Na_p_ | Max. cond. of persistent Na channel | Soma | 0, 10^-4^ | S∙cm^-2^ |
| I_h_ | Max. cond. of Ih channel | Soma, dendrites | 5x10^-6^, 2x10^-5^ | S∙cm^-2^ |
| R_a_ | Axial resistance | Axon | 50, 100 | 𝛀∙cm |
|  |  | Soma, dendrites | 100, 400 | 𝛀∙cm |
| 𝛾_Ca_ | Fraction of free calcium | Soma, dendrites | 0.1, 1 |  |
| 𝜏_Ca_ | Calcium dynamics time constant | Soma, dendrites | 20, 1000 | Ms |
| E_pas_ | Equilibrium potential of passive channels | Axon | -80, -50 | mV |
|  |  | Soma, dendrites | -80, -55 | mV |
| g_pas_ | Max. cond. of passive channels | Axon | 2x10^-5^, 2x10^-4^ | S∙cm^-2^ |
|  |  | Soma, dendrites | 5x10^-6^, 10^-4^ | S∙cm^-2^ |

Supplementary Table 2. Parameter names as shown in Figs 3 and S1 with corresponding channel description, location on the cell morphology and allowed bounds of variation during optimization.
